# Labelled Interpolation Systems for Hyper-Resolution, Clausal, and Local Proofs

**DOI:** 10.1007/s10817-016-9364-6

**Published:** 2016-02-16

**Authors:** Matthias Schlaipfer, Georg Weissenbacher

**Affiliations:** TU Wien, Vienna, Austria

**Keywords:** Craig interpolation, Satisfiability checking, Resolution

## Abstract

Craig’s interpolation theorem has numerous applications in model checking, automated reasoning, and synthesis. There is a variety of interpolation systems which derive interpolants from refutation proofs; these systems are ad-hoc and rigid in the sense that they provide exactly one interpolant for a given proof. In previous work, we introduced a parametrised interpolation system which subsumes existing interpolation methods for propositional resolution proofs and enables the systematic variation of the logical strength and the elimination of non-essential variables in interpolants. In this paper, we generalise this system to propositional hyper-resolution proofs as well as clausal proofs. The latter are generated by contemporary SAT solvers. Finally, we show that, when applied to local (or split) proofs, our extension generalises two existing interpolation systems for first-order logic and relates them in logical strength.

## Introduction

Craig interpolation [14] has proven to be an effective heuristic in applications such as model checking, where it is used as an approximate method for computing invariants of transition systems [39, 54], and synthesis, where interpolants represent deterministic implementations of specifications given as relations [31]. The intrinsic properties of interpolants enable concise abstractions in verification and smaller circuits in synthesis. Intuitively, stronger interpolants provide more precision [29, 46], and interpolants with fewer variables lead to smaller designs [7, 31]. However, interpolation is mostly treated as a black box, leaving no room for a systematic exploration of the solution space. In addition, the use of different interpolation systems complicates a comparison of their interpolants. We present a novel framework which generalises a number of existing interpolation techniques and supports a systematic variation and comparison of the generated interpolants.

### Contributions

We present a novel *parametrised* interpolation system which extends our previous work on propositional interpolation [16].

- The extended system supports hyper-resolution (see Sect. 3) and allows for systematic variation of the logical strength (with an additional degree of freedom over [16]) and the elimination of non-essential literals [15] in interpolants.
- We generalise (in Sect. 4) our interpolation system for hyper-resolution steps to clausal refutations generated by contemporary SAT solvers such as PicoSAT [5], allowing us to avoid the generation of intermediate interpolants.
- When applied to local (or split) proofs [30], the extended interpolation system generalises the existing interpolation systems for first-order logic presented in [32, 55] and relates them in logical strength (Sect. 5).

This paper is an extended version of [56], and includes novel results on interpolation for clausal proofs and empirical results (see Sect. 4).

## Background

This section introduces our notation (Sect. 2.1) and restates the main results of our previous paper on labelled interpolation systems [16] in Sect. 2.2.

### Formulae and Proofs

In our setting, the term *formula* refers to either a propositional logic formula or a formula in standard first-order logic.

#### Propositional Formulae

We work in the standard setting of propositional logic over a set *X* of propositional variables, the logical constants ${\mathsf {T}}$ and ${\mathsf {F}}$ (denoting true and false, respectively), and the standard logical connectives $\wedge$, $\vee$, $\Rightarrow$, and $\lnot$ (denoting conjunction, disjunction, implication, and negation, respectively).

Moreover, let $\mathtt {Lit}_X = \{x, \overline{x}\,\vert \, x \in X\}$ be the set of literals over *X*, where $\overline{x}$ is short for $\lnot x$. We write ${\mathrm {var}}(t)$ for the variable occurring in the literal $t\in \mathtt {Lit}_X$. A clause *C* is a set of literals. The empty clause $\Box$ contains no literals and is used interchangeably with ${\mathsf {F}}$. The disjunction of two clauses *C* and *D* is their union, denoted $C \vee D$, which is further simplified to $C \vee t$ if *D* is the singleton $\{t\}$. In clauses, we sometimes omit the disjunction $\vee$ to save space. A propositional formula in conjunctive normal form (CNF) is a conjunction of clauses, also represented as a set of clauses.

#### First-Order Logic

The logical connectives from propositional logic carry over into first-order logic. We fix an enumerable set of variables, function and predicate symbols over which formulae are built in the usual manner. The *vocabulary* of a formula *A* is the set of its function and predicate symbols. ${\mathcal {L}}({A})$ refers to the set of well-formed formulae which can be built over the vocabulary of *A*.

Variables may be universally $(\forall )$ or existentially $(\exists )$ quantified. A formula is *closed* if all its variables are quantified and *ground* if it contains no variables. As previously, conjunctions of formulae are also represented as sets.

Given a formula *A* in either first-order or propositional logic, we use ${\mathrm {Var}}(A)$ to denote the set of free (unquantified) variables in *A*.

#### Inference Rules and Proofs

We write $A_1, \ldots , A_n\models A$ to denote that the formula *A* holds in all models of $A_1, \ldots , A_n$ (where $n\ge 0$). An inference rule

1 $$\begin{aligned} \frac{A_1 \quad \cdots \quad A_n}{A} \end{aligned}$$

associates zero or more *premises* (or *antecedents*) $A_1, \ldots , A_n$ with a *conclusion* *A*. The inference rule (1) is *sound* if $A_1, \ldots , A_n\models A$ holds. A (sound) inference system ${\mathcal {I}}$ is a set of (sound) inference rules.

The propositional *resolution* rule $({\mathrm {Res}})$, for example, is a sound inference rule stating that an assignment satisfying the clauses $C \vee x$ and $D \vee \overline{x}$ also satisfies $C \vee D$. The clauses $C \vee x$ and $D \vee \overline{x}$ are the *antecedents*, *x* is the *pivot*, and the conclusion $C \vee D$ is called the *resolvent*. ${\mathrm {Res}}(C,D,x)$ denotes the resolvent of *C* and *D* with the pivot *x*.

##### **Definition 1**

(*Proof*) A *proof* (or derivation) *P* in an inference system ${\mathcal {I}}_P$ is a directed acyclic graph $(V_P, E_P, \ell _P, \mathtt {s}_P)$, where $V_P$ is a set of vertices, $E_P$ is a set of edges, $\ell _P$ is a function mapping vertices to formulae, and $\mathtt {s}_P \in V_P$ is the sink vertex. An *initial vertex* has in-degree 0. All other vertices are *internal* and have in-degree $\ge 1$. The sink has out-degree 0. Each internal vertex *v* with edges $(v_1, v), \ldots , (v_m, v) \in E_P$ is associated with an inference rule ${\mathsf {Inf}}\in {\mathcal {I}}_P$ with antecedents $\ell _P(v_1), \ldots , \ell _P(v_m)$ and conclusion $\ell _P(v)$.

The subscripts above are dropped if clear. A vertex $v_i$ in *P* is a *parent* of $v_j$ if $(v_i,v_j) \in E_P$. A proof *P* is a *refutation* if $\ell _P(\mathtt {s}_P)={\mathsf {F}}$. Let *A* and *B* be conjunctive formulae. A refutation *P* of an unsatisfiable formula $A\wedge B$ is an (*A*, *B*)-*refutation* (i.e., for each initial vertex $v\in V_P$, $\ell _P(v)$ is a conjunct of *A* or a conjunct of *B*). A proof is *closed* (*ground*, respectively) if $\ell _P(v)$ is closed (ground) for all $v\in V_P$.

In the following, we use the propositional resolution calculus to instantiate Definition 1.

##### **Definition 2**

(*Resolution Proof*) A *resolution proof* *R* is a proof in the inference system comprising only the resolution rule ${\mathsf {Res}}$. Consequently, $\ell _R$ maps each vertex $v\in V_R$ to a clause, and all internal vertices have in-degree 2. Let ${ piv }_R$ be the function mapping internal vertices to pivot variables. For an internal vertex *v* and $(v_1, v), (v_2, v) \in E_R$, $\ell _R(v) = {\mathrm {Res}}(\ell _R(v_1), \ell _R(v_2),{ piv }_R(v))$.

Note that the value of $\ell _R$ at internal vertices is determined by that of $\ell _R$ at initial vertices and the pivot function ${ piv }_R$. We write $v^+$ for the parent of *v* with ${ piv }(v)$ in $\ell (v^+)$ and $v^-$ for the parent with $\lnot { piv }(v)$ in $\ell (v^-)$.

A resolution proof *R* is a *resolution refutation* if $\ell _R(\mathtt {s}_R) = \Box$.

### Interpolation Systems and Labelling Functions

There are numerous variants and definitions of Craig’s interpolation Theorem [14]. We use the definition of a Craig interpolant introduced by McMillan [39]:

#### **Definition 3**

(*Interpolant*) A Craig *interpolant* for a pair of formulae (*A*, *B*), where $A \wedge B$ is unsatisfiable, is a formula *I* whose free variables, function and predicate symbols occur in both *A* and *B*, such that $A \Rightarrow I$ and $B \Rightarrow \lnot I$ hold.

Craig’s interpolation theorem guarantees the existence of such an interpolant for unsatisfiable pairs of formulae (*A*, *B*) in first order logic. Consequently, it also holds in the propositional setting, where the conditions of Definition 3 reduce to $A\Rightarrow I$, $B\Rightarrow \lnot I$, and ${\mathrm {Var}}(I) \subseteq {\mathrm {Var}}(A) \cap {\mathrm {Var}}(B)$.

#### *Example 1*

**Fig. 1:**
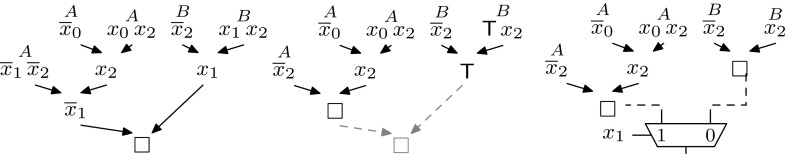
The interpolant $\overline{x}_1$ acts as a “separator” for the resolution refutation

Let $A=(\overline{x}_0)\wedge (x_0\vee x_2)\wedge (\overline{x}_1\vee \overline{x}_2)$ and $B=(\overline{x}_2)\wedge (x_1\vee x_2)$. Then $I=\overline{x}_1$ is an interpolant for (*A*, *B*). Intuitively, $\overline{x}_1$ interpolant acts as a “separator” for the underlying refutation proof (the leftmost proof in Fig. 1). By setting $\overline{x}_1$ to ${\mathsf {F}}$ we obtain a refutation of the *A*-partition, as illustrated in Fig. 1. Similarly, setting $\overline{x}_1$ to ${\mathsf {T}}$ yields a refutation for *B*—the interpolant can be understood as a multiplexer. Equivalently, *I* is ${\mathsf {T}}$ if *A* is ${\mathsf {T}}$, and $\lnot I$ is ${\mathsf {T}}$ if *B* is ${\mathsf {T}}$.

Numerous techniques to construct interpolants have been proposed (c.f. Sect. 6). In particular, there is a class of algorithms that derive interpolants from proofs; the first such algorithm for the sequent calculus is presented in Maehara’s constructive proof [37] of Craig’s theorem. In this paper, we focus on *interpolation systems* that construct an interpolant from an (*A*, *B*)-refutation by mapping the vertices of a resolution proof to a formula called the *partial interpolant*.

Formally, an interpolation system ${\mathsf {Itp}}$ is a function that given an (*A*, *B*)-refutation *R* yields a function, denoted ${\mathsf {Itp}}(R,A,B)$, from vertices in *R* to formulae over ${\mathrm {Var}}(A)\cap {\mathrm {Var}}(B)$. An interpolation system is *correct* if for every (*A*, *B*)-refutation *R* with sink $\mathtt {s}$, it holds that ${\mathsf {Itp}}(R,A,B) (\mathtt {s})$ is an interpolant for (*A*, *B*). We write ${\mathsf {Itp}}(R)$ for ${\mathsf {Itp}}(R,A,B) (\mathtt {s})$ when *A* and *B* are clear. Let *v* be a vertex in an (*A*, *B*)-refutation *R*. The pair $(\ell (v), {\mathsf {Itp}}(R,A,B) (v))$ is an *annotated clause* and is written $\ell (v)\;[{\mathsf {Itp}}(R,A,B) (v)]$ in accordance with [40].

In the following, we review the labelled interpolation systems we introduced in [16]. Labelled interpolation generalises several existing propositional interpolation systems presented by Huang [28], Krajíček  [33], Pudlák  [42], and McMillan [39]. A distinguishing feature of a labelled interpolation system is that it assigns an individual label  to *each literal* in the resolution refutation.
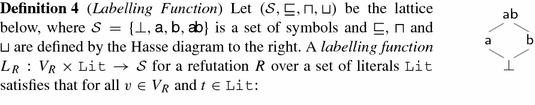

1. $$L_R(v,t) = \bot $$ iff $$t \notin \ell _R(v)$$
2. $L_R(v,t) = L_R(v_1,t) \sqcup \cdots \sqcup L_R(v_m,t)$ for an internal vertex *v*, its parents $\{v_1, \ldots , v_m\}$, and literal $t \in \ell _R(v)$.

Due to Condition (2) above, the labels of literals at initial vertices completely determine the labelling function for literals at internal vertices. The following condition ensures that a labelling function respects the *locality* of a literal *t* in accordance with (*A*, *B*). A literal *t* is *A*-*local* and therefore labelled ${\textsf {a}}$ if ${\mathrm {var}}(t)\in {\mathrm {Var}}(A){\setminus }{\mathrm {Var}}(B)$. Conversely, *t* is *B*-*local* and therefore labelled ${\textsf {b}}$ if ${\mathrm {var}}(t) \in {\mathrm {Var}}(B){\setminus }{\mathrm {Var}}(A)$. Literals *t* for which ${\mathrm {var}}(t)\in {\mathrm {Var}}(A)\cap {\mathrm {Var}}(B)$ are *shared* and can be labelled ${\textsf {a}}$, ${\textsf {b}}$, or  (which generalises existing interpolation systems).

#### **Definition 5**

(*Locality*) A labelling function *L* for an (*A*, *B*)-refutation *R* *preserves locality* if for any initial vertex *v* and literal *t* in *R*

1. ${\textsf {a}} \sqsubseteq L(v,t)$ implies that ${\mathrm {var}}(t)\in {\mathrm {Var}}(A)$, and
2. $${\textsf {b}} \sqsubseteq L(v,t)$$ implies that $${\mathrm {var}}(t)\in {\mathrm {Var}}(B)$$.

For a given labelling function *L*, we define the downward *projection* of a clause at a vertex *v* with respect to ${\textsf {c}} \in {\mathcal {S}}$ as $\ell (v)\downharpoonright _{{\textsf {c}},L} \mathop {=}\limits ^{\tiny def }\{t \in \ell (v)\,\vert \, L(v,t) \sqsubseteq {\textsf {c}} \}$ and the upward projection $\ell (v)\upharpoonright _{{\textsf {c}},L}$ as $\ell (v)\upharpoonright _{{\textsf {c}},L} \mathop {=}\limits ^{\tiny def }\{t \in \ell (v)\,\vert \, {\textsf {c}} \sqsubseteq L(v,t)\}$. The subscript *L* is omitted if clear from the context.

#### **Definition 6**

**Fig. 2:**
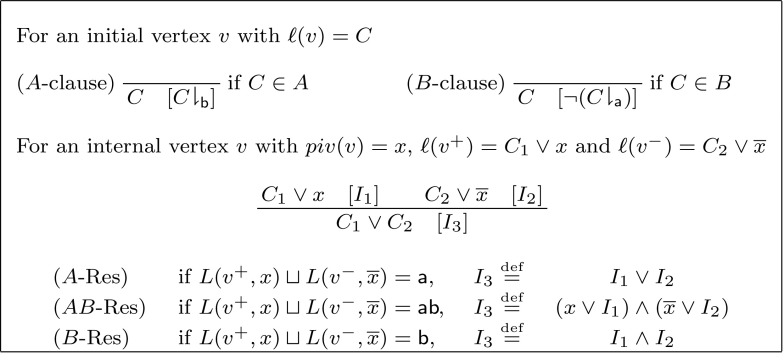
Labelled interpolation system for resolution proofs

(*Labelled Interpolation System for Resolution*) Let *L* be a locality preserving labelling function for an (*A*, *B*)-refutation *R*. The labelled interpolation system ${\mathsf {Itp}}(L)$ maps vertices in *R* to partial interpolants as defined in Fig. 2.

Labelling functions provide control over the interpolants constructed from a resolution proof. Firstly, labelled interpolation systems support the elimination of *non-essential* (*peripheral* [50], respectively) variables from interpolants [15]. Secondly, labelled interpolation systems—and their respective interpolants—are ordered by logical strength. A labelled interpolation system ${\mathsf {Itp}}(L)$ is *stronger than* ${\mathsf {Itp}}(L^{\prime })$ if for all refutations *R* (for which *L* and $L^{\prime }$ are locality preserving labelling functions), ${\mathsf {Itp}}(L,R) \Rightarrow {\mathsf {Itp}}(L^{\prime },R)$. The partial order $\preceq$ on labelling functions (first introduced in [16]) guarantees an ordering in strength:
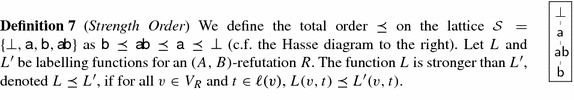


Theorem 2 in [16] shows that if *L* is a stronger labelling function than $L^{\prime }$, the interpolant obtained from ${\mathsf {Itp}}(L)$ logically implies the one obtained from ${\mathsf {Itp}}(L^{\prime })$.

## Interpolation for Hyper-Resolution

In this section, we extend labelled interpolation systems to a richer inference system, in particular, the inference system comprising (propositional) *hyper-resolution* [43]. Hyper-resolution is a condensation of a derivation consisting of several resolutions and avoids the construction of intermediate clauses. Hyper-resolution has several applications in propositional satisfiability checking, such as pre-processing [21] of formulae or as an integral part of the solver (e.g., [2]).

*Positive* hyper-resolution combines a single clause (called the *nucleus*) containing *n* negative literals $\overline{x}_1, \ldots , \overline{x}_n$ and *n* *satellite* clauses each of which contains one of the corresponding non-negated literals $x_i$ (where $1\le i\le n$):In *negative* hyper-resolution the roles of ${x}_i$ and $\overline{x}_i$ are exchanged.

### **Definition 8**

(*Hyper-Resolution Proof*) A *hyper-resolution proof* *R* is a proof using only the inference rule ${\mathsf {HyRes}}$. Accordingly, $\ell _R$ maps each vertex $v\in V_R$ to a clause, and all internal vertices have in-degree $\ge 2$. Each internal vertex *v* has $n\ge 1$ parents $v^+_1, \ldots , v^+_n$ such that $\ell _R(v^+_i)=C_i\vee x_i$ and one parent $v^-$ with $\ell _R(v^-)=\overline{x}_1\vee \cdots \vee \overline{x}_n\vee D$, and consequently, $\ell _R(v)=\bigvee _{i=1}^n C_i\vee D$.

The definition of labelling functions (Definition 4) readily applies to hyper-resolution proofs. Note that $\preceq$ is not a total order on labelling functions. Lemma 1 (a generalisation of Lemma 3 in [16] to hyper-resolution proofs) enables a comparison of labelling functions based solely on the values at the *initial* vertices.

### **Lemma 1**

Let *L* and $L^{\prime }$ be labelling functions for an (*A*, *B*)-refutation *R*. If $L(v,t) \preceq L^{\prime }(v,t)$ for all initial vertices *v* and literals $t \in \ell (v)$, then $L \preceq L^{\prime }$.

**Fig. 3:**
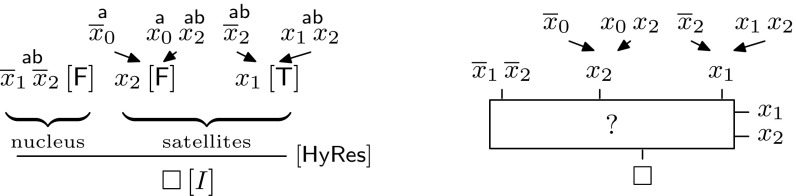
Generalising labelled interpolation to hyper-resolution

A proof of Lemma 1 is given in Appendix 1. In the following, we generalise labelled interpolation systems to hyper-resolution. The underlying intuition is to replace the multiplexer in the case $AB$-Res in Definition 6 with a general multiplexer controlled by the pivot literals of the hyper-resolution step. This idea is illustrated in Fig. 3 for the proof in Example 1 and formalised in the following definition:

### **Definition 9**

**Fig. 4:**
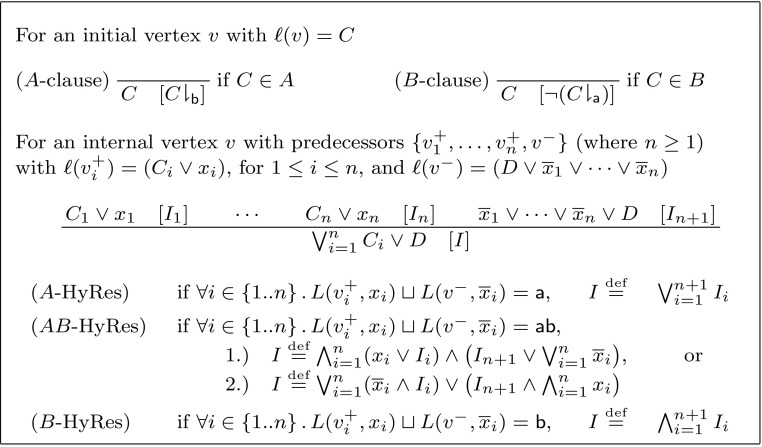
Labelled interpolation system for hyper-resolution proofs

(*Labelled Interpolation System for Hyper-Resolution*) Let *L* be a locality preserving labelling function for an (*A*, *B*)-refutation *R*, where *R* is a hyper-resolution proof. The labelled interpolation system ${\mathsf {Itp}}(L)$ maps vertices in *R* to partial interpolants as defined in Fig. 4.

The interpolation system leaves us a choice for internal nodes $AB$-HyRes. We will use ${\mathsf {Itp}}_1$ (${\mathsf {Itp}}_2$, respectively) to refer to the interpolation system that always chooses case 1 (case 2, respectively). Note furthermore that Definitions 6 and 9 are equivalent in the special case where $n=1$.

### *Remark 1*

Note that unlike the interpolation system for ordinary resolution proofs presented in Definition 6, ${\mathsf {Itp}}$ is not total for hyper-resolution proofs: the case split requires the pivots of the hyper-resolution step to be uniformly labelled, i.e., the rules $A$-HyRes, $AB$-HyRes, and $B$-HyRes require $L(v^+_i, x_i)\sqcup L(v^-, \overline{x}_i)$ to be ${\textsf {a}}$, , or ${\textsf {b}}$, respectively, for all $i\in \{1, \ldots , n\}$. This limitation is addressed in Sect. 4.1.

In the following we present a conditional correctness result:

### **Theorem 1**

(Correctness) For any (*A*, *B*)-refutation *R* (where *R* is a hyper-resolution proof) and locality preserving labelling function *L*, ${\mathsf {Itp}}(L,R)$ (if defined) is an interpolant for (*A*, *B*).

The proof of Theorem 1 (given in Appendix 1) establishes that for each vertex $v\in V_R$ with $\ell _R(v)=C$ and $I={\mathsf {Itp}}(L,R) (v)$, the following conditions hold:

- $$A\wedge \lnot (C\upharpoonright _{{\textsf {a}},L})\Rightarrow I$$,
- $$B\wedge \lnot (C\upharpoonright _{{\textsf {b}},L})\Rightarrow \lnot I$$, and
- $$\text {Var}(I)\subseteq \text {Var}(A)\cap \text {Var}(B)$$.

For $\ell _R(\mathtt {s})=\Box$, this establishes the correctness of the system.

We emphasise that Theorem 1 does not constrain the choice for the case $AB$-HyRes. Since both ${\mathsf {Itp}}_1(L,R)$ and ${\mathsf {Itp}}_2(L,R)$ satisfy the conditions above, this choice does not affect the correctness of the interpolation system. In fact, it is valid to *mix* both systems by defining a choice function $\chi : V_R\rightarrow \{1,2\}$ which determines which interpolation system is chosen at each internal node. We use ${\mathsf {Itp}}_{\chi }(L,R)$ to denote the resulting interpolation system. This modification, however, may have an impact on the logical strength of the resulting interpolant.

### **Theorem 2**

Let the hyper-resolution proof *R* be an (*A*, *B*)-refutation and *L* be a locality preserving labelling function. Moreover, let ${\mathsf {Itp}}_{\chi }(L,R)$ and ${\mathsf {Itp}}_{\chi ^{\prime }}(L,R)$ be labelled interpolation systems (defined for *L*, *R*) with the choice functions $\chi$ and $\chi ^{\prime }$, respectively. Then ${\mathsf {Itp}}_{\chi }(L,R)\Rightarrow {\mathsf {Itp}}_{\chi ^{\prime }}(L,R)$ if $\chi (v)\le \chi ^{\prime }(v)$ for all internal vertices $v\in V_R$.

### *Proof sketch*

This follows (by structural induction over *R*) from

$$\begin{aligned} \left( {\bigwedge }_{i=1}^n (x_i \vee I_i) \wedge \left( I_{n+1}\vee {\bigvee }_{i=1}^n\overline{x}_i \right) \right) \Rightarrow \left( {\bigvee }_{i=1}^n (\overline{x}_i \wedge I_i) \vee \left( I_{n+1}\wedge {\bigwedge }_{i=1}^n x_i \right) \right) . \end{aligned}$$

$$\square $$

Note that the converse implication does not hold; a simple counterexample for an internal vertex with $n=2$ is the assignment $x_1=x_2={\mathsf {F}}$, $I_1={\mathsf {T}}$, and $I_2=I_3={\mathsf {F}}$.

The final theorem in this section extends the result of Theorem 2 in [16] to hyper-resolution proofs:

### **Theorem 3**

If *L* and $L^{\prime }$ are labelling functions for an (*A*, *B*)-refutation *R* (*R* being a hyper-resolution proof) and $L \preceq L^{\prime }$ such that ${\mathsf {Itp}}_i(L,R)$ as well as ${\mathsf {Itp}}_i(L^{\prime },R)$ are defined, then ${\mathsf {Itp}}_i(L,R) \Rightarrow {\mathsf {Itp}}_i(L^{\prime },R)$ (for a fixed $i\in \{1,2\}$).

The proof of Theorem 3, provided in Appendix 1, is led by structural induction over *R*. For any vertex *v* in *R*, let $I_v$ and $I^{\prime }_v$ be the partial interpolants due to ${\mathsf {Itp}}_i(L,R)$ and ${\mathsf {Itp}}_i(L^{\prime },R)$, respectively. We show that  for all vertices *v*, establishing $I_v\Rightarrow I_v^{\prime }$ for the sink to show that ${\mathsf {Itp}}_i(L,R) \Rightarrow {\mathsf {Itp}}_i(L^{\prime },R)$.

Theorems 2 and 3 enable us to fine-tune the strength of interpolants, since the sets of all labelling and choice functions ordered by $\preceq$ and $\le$, respectively, form complete lattices (c.f. [16, Theorem 3]). Finally, we remark that the Theorems 2 and 3 are orthogonal. The former fixes the labelling function *L*, whereas the latter fixes the choice function $\chi$.

## Interpolation for Clausal Proofs

Contemporary SAT solvers such as MiniSAT [17] and PicoSAT [5] are based on conflict-driven clause learning (CDCL) [49]. The CDCL algorithm avoids the repeated exploration of conflicting variable assignments by caching the causes of failures in the form of learned clauses. To this end, the solver stores assignments (decisions) and their implications in an *implication graph*, from which it derives learned clauses in case of a conflict. We refrain from providing a description of CDCL, since numerous excellent expositions are available (e.g., [6, 34]). The following example, borrowed from [38], illustrates the construction of resolution proofs in CDCL solvers.

### *Example 2*

**Fig. 5:**
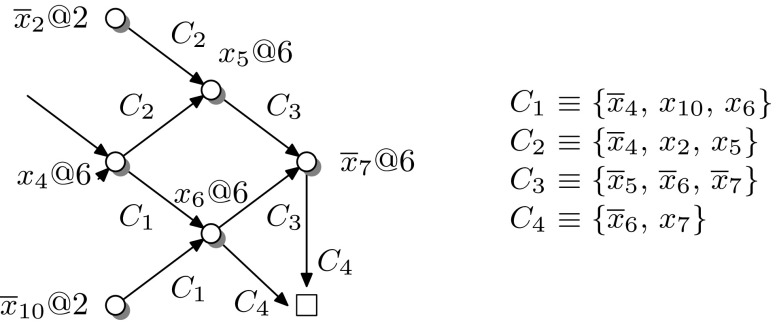
Implication graph and conflict analysis

Figure 5 shows a partial implication graph for the clauses $(\overline{x}_4\,{x}_{10}\,{x}_6)$, $(\overline{x}_4\,{x}_2\,{x}_5)$, $(\overline{x}_5\,\overline{x}_{6}\,\overline{x}_7)$, and $(\overline{x}_6\,{x}_7)$. Nodes represent assignments (annotated with the corresponding decision level, e.g., $\overline{x}_{10}@2$ indicates that $x_{10}$ was assigned ${\mathsf {F}}$ at level 2) and each edge represents an implication deriving from a clause in which all but one literal is assigned under the current assignment. The final node $\Box$ indicates a conflict under the current assignment, and its incoming edges are annotated with the conflicting clause $C_4$. This conflict stems from the fact that $C_4$ disagrees with $C_1$ and $C_3$ on the implied literals $\overline{x}_6$ and $x_7$, respectively. By subsequently resolving on the conflicting literals, we obtain

$$\begin{aligned} C_5={\mathrm {Res}}(C_4,C_3,x_7)=\{\overline{x}_5,\,\overline{x}_6\}\quad \text {and}\quad C_6={\mathrm {Res}}(C_5,C_1,x_6)=\{\overline{x}_4,\,\overline{x}_5,\,{x}_{10}\}. \end{aligned}$$

The clause $C_6$ disagrees with $C_2$ on the implied literal $x_5$. The resolvent of these clauses is $C_7={\mathrm {Res}}(C_6,C_2,x_5)=\{{x}_2,\,\overline{x}_4,\,{x}_{10}\}$. $C_7$ contains a single literal $(x_4)$ assigned at decision level 6 while still conflicting with the current partial assignment. Accordingly, reverting the decision $x_4$ at level 6 and adding $C_7$ as learned clause prevents the solver from revisiting this part of the search space.

The learned clause in Example 2 is a consequence of clauses of the original instance and previously learned clauses. Each learned clause is the conclusion of a chain of resolution steps.

### **Definition 10**

(*Chain*) A (resolution) *chain* of length *n* is a tuple consisting of an input clause $D_0$ and an ordered sequence of clause-pivot pairs $\langle C_i, x_i\rangle$ (where $1\le i\le n$). The final resolvent $D_n$ of a resolution chain is defined inductively as $D_i={\mathrm {Res}}(D_{i-1},C_i, x_i)$.

A resolution chain generated by a CDCL solver has the following properties [4]:

- Regularity: each pivot variable is resolved upon at most once in the chain.
- Linearity: each intermediate clause $D_i$ $(1\le i\le n)$ in a chain is obtained by deriving $D_{i-1}$ with an initial clause $C_j$ ($2\le j\le n$) or with a previously derived clause $D_k$ ($k<i-1$).
- Tree-likeness: each derived clause is used exactly once in the chain.

A resolution derivation with these properties is called trivial [4]. For reasons of performance, proof-logging solvers discard all intermediate resolvents generated during the construction of a conflict clause and retain only resolution chains. Clausal proofs [22, 25] and proofs stored in the TraceCheck-format^1^ moreover omit the pivot literals as well as the order of the resolution steps, recording only the unordered set of clauses $D_0, C_1,\ldots , C_n$ for each resolution chain.

If $D_0$ is a nucleus and $C_1, \ldots , C_n$ are suitable satellites, the chain can be replaced by a hyper-resolution step assuming its conclusion $D_n$ satisfies the ${\mathrm {HyRes}}$ rule. In general, this may not be the case: $D_0=\{x_1,\,x_2\}, C_1=\{\overline{x}_2,\,x_3\}, C_2=\{\overline{x}_3,\,x_4\}$ is a valid resolution chain (with conclusion $\{x_1,x_4\}$) that does not match the antecedents ${\mathrm {HyRes}}$ rule.

To address this problem, we introduce a more general inference rule which requires the existence of a resolution chain matching its premises and conclusion as a side condition. Each of the *n* premises contains a non-empty (sub-)set of pivot literals $P_i$ which occur in opposite phase in the other clauses of the premise. The clause learning algorithm illustrated in Example 2 results in resolution chains that satisfy the following properties:

- The pivot literals $\bigcup _{i=1}^n P_i$ do not occur in the conclusion of the chain.
*Remark 2* The algorithm resolves upon pivot literals that are implied but not yet assigned at the respective node in the implication graph. Accordingly, the clauses preceding the node in the implication graph cannot contain the implied literal, since they would otherwise not be unit. Therefore, a pivot literal, once resolved, is never re-introduced in a resolution chain.
- The conjunction $\bigwedge _{i=1}^n P_i$ is unsatisfiable (guaranteed by the existence of a resolution chain).

These properties are reflected in the following inference rule:

### **Definition 11**

(TraceCheck  *Resolution*) Let $D_1, \ldots , D_n$ be an (unordered) set of clauses. Let $P_i\mathop {=}\limits ^{\tiny def }\{t\in D_i\,\vert \,\exists j\,\cdot \,1\le j\le n\wedge j\ne i\wedge \overline{t}\in D_j\}$ and $C_i\mathop {=}\limits ^{\tiny def }D_i{\setminus } P_i$. If there exists a resolution chain $D_1, \langle D_2, x_2\rangle , \ldots , \langle D_n,x_n\rangle$ with conclusion $\bigvee _{i=1}^n C_i$ then

$$\begin{aligned} \frac{(C_1 \vee P_1) \quad \cdots \quad (C_n\vee P_n)}{\bigvee _{i=1}^n C_i} \quad {[{\mathsf {TCRes}}]} \end{aligned}$$

Analogously to Definition 8, we introduce the notion of a clausal proof.

### **Definition 12**

(*Clausal Proof*) A clausal proof *R* is a proof using only the inference rule ${\mathsf {TCRes}}$. Accordingly, $\ell _R$ maps each vertex $v \in V_R$ to a clause and every internal vertex *v* has $n \ge 2$ parents $v_1, \ldots , v_n$ such that $\ell _R(v_i) = C_i \vee P_i$ (as in Definition 11). Consequently, $\ell _R(v)=\bigvee _{i=1}^n C_i$.

The following definition extends the interpolation system for hyper-resolution proofs presented in Sect. 3 to clausal proofs.

### **Definition 13**

**Fig. 6:**
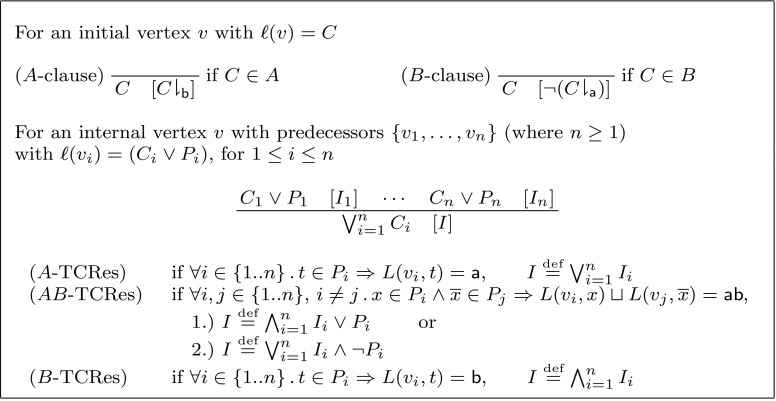
Labelled interpolation system for clausal proofs

(*Labelled Interpolation System for Clausal Proofs*) Let *L* be a locality preserving labelling function for an (*A*, *B*)-refutation *R*, where *R* is a clausal proof. The labelled interpolation system ${\mathsf {Itp}}(L)$ maps vertices in *R* to partial interpolants as defined in Fig. 6.

Note that the interpolation system in Definition 13 is a generalisation of the interpolation system for hyper-resolution (Definition 9). Its correctness is established using a similar argument as used for Theorem 1. The proof of the following theorem is provided in Appendix 1.

### **Theorem 4**

(Correctness) For any (*A*, *B*)-refutation *R* (where *R* is a clausal proof) and locality preserving labelling function *L*, ${\mathsf {Itp}}(L,R)$ (if defined) is an interpolant for (*A*, *B*).

The results of Theorems 2 and 3 can be generalised to clausal proofs in a straight-forward manner. We omit the discussion of the details.

### Splitting and Reordering Resolution Chains

Just like the interpolation system for hyper-resolution proofs, the interpolation system in Definition 13 has the deficiency that the function ${\mathsf {Itp}}(L)$ is not total: there are labelling functions *L* for which the result of ${\mathsf {Itp}}(L)$ is undefined. This problem arises whenever the pivots in a TraceCheck resolution step are not uniformly labelled, and therefore none of the rules in Fig. 6 is applicable.

**Fig. 7:**
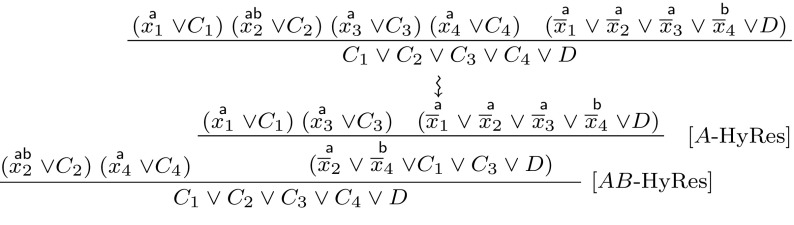
Splitting hyper-resolution steps

Instead of adapting the interpolation system, we address the problem by splitting the corresponding resolution chains. A single chain can be split into two consecutive chains, with the final resolvent of the first acting as the input clause of the second, without affecting the final result. By splitting resolution steps whose pivots are not uniformly labelled we can *always* generate a labelled refutation for which ${\mathsf {Itp}}$ is a total function. The example in Fig. 7 illustrates this transformation for a single hyper-resolution step.

Each hyper-resolution or TraceCheck resolution step may need to be rewritten into several subsequent uniformly labelled steps, thus changing the proof structure. Note that the results on the relative strength of interpolants in Sect. 3 naturally only apply if both proofs have the same structure. The effect of the order of resolution steps on the strength of interpolants is discussed in [16, Section 5.2] and exceeds the scope of this paper.

The number of resolution steps resulting from splitting depends on the order of the pivots in the given resolution chain, as demonstrated in the following example.

#### *Example 3*

**Fig. 8:**
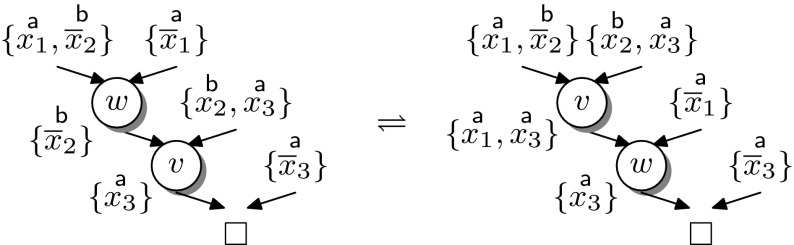
Reordering resolution chains

Figure 8 shows two resolution chains (presented as trivial resolution proofs). In the left proof, the order of the pivots is $\mathop {x_1}\limits ^{{\textsf {a}}}$, $\mathop {x_2}\limits ^{{\textsf {b}}}$, $\mathop {x_3}\limits ^{{\textsf {a}}}$, necessitating *two* splits to obtain a uniform labelling of the pivots. The proof to the right corresponds to a similar resolution chain in which the first two resolution steps have been swapped. The resulting split yields the following two TraceCheck resolution steps:

$$\begin{aligned} \frac{(\mathop {x_1}\limits ^{{\textsf {a}}}\vee \mathop {\overline{x}_2}\limits ^{{\textsf {b}}})\quad (\mathop {x_2}\limits ^{{\textsf {b}}}\vee \mathop {x_3}\limits ^{{\textsf {a}}})}{(\mathop {x_1}\limits ^{{\textsf {a}}}\vee \mathop {x_3}\limits ^{{\textsf {a}}})} \quad {[B\text {-}{\mathrm {TCRes}}]} \quad \frac{(\mathop {x_1}\limits ^{{\textsf {a}}}\vee \mathop {x_3}\limits ^{{\textsf {a}}})\quad (\mathop {\overline{x}_1}\limits ^{{\textsf {a}}})\quad (\mathop {\overline{x}_3}\limits ^{{\textsf {a}}})}{\Box } \quad {[A\text {-}{\mathrm {TCRes}}]} \end{aligned}$$

Accordingly, the interpolation system ${\mathsf {Itp}}(L)$ is applicable to the corresponding clausal proof.

**Fig. 9:**
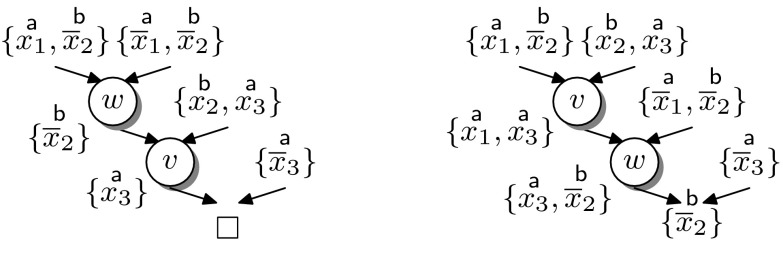
Reordering in the presence of merge literals may invalidate the resolution chain

Example 3 shows that reordering the resolution steps in a chain can result in fewer uniformly labelled TraceCheck resolution steps. A *swap* $(\rightleftharpoons )$ of two subsequent resolution steps, formally defined in [16, Def. 10] and illustrated in Fig. 8, is allowed whenever it does not change the conclusion of the resolution chain. In the presence of merge literals [1] (i.e., literals $t \in \ell (v)$ such that $t \in \ell (v^+)$ and $t \in \ell (v^-)$) this is not guaranteed [16], as illustrated in Fig. 9.

The final resolvent of a chain may depend on the order of the ordinary resolution steps: literal $\overline{x}_2$ is re-introduced after being eliminated in the modified chain, while it is *merged* and eliminated once and for all in the original chain.

In the absence of merge literals, this issue does not arise. For this reason, [56] prohibits merge literals in resolution chains (in addition to requiring that the premises match the ${\mathrm {HyRes}}$ rule). While this guarantees that a any permutation of the clause-pivot sequence still represents a valid resolution chain and leaves the final resolvent unaffected (an immediate consequence of [16, Lemma 4]), the requirement is overly restrictive. In the following, we discuss conditions under which reordering does not invalidate the proof even in the presence of merge literals.

**Fig. 10:**
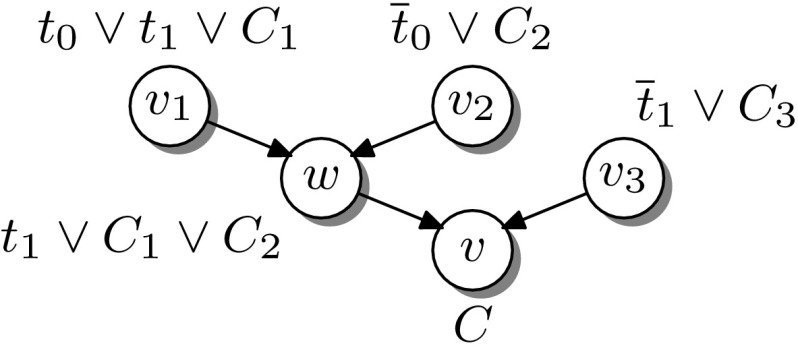
Proof *R*

**Fig. 11:**
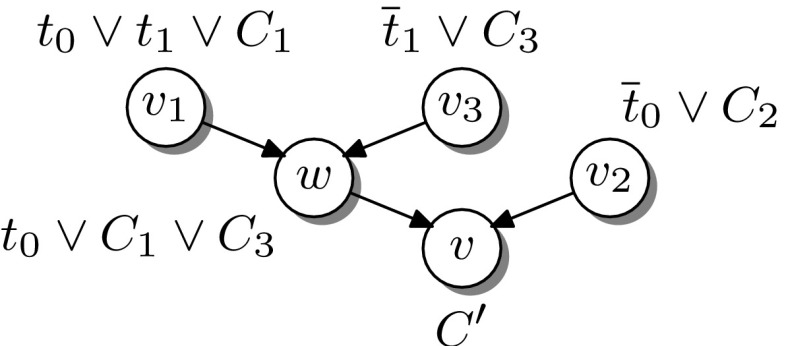
Graph $$R^{\prime } \mathop {=}\limits ^{\tiny def }{R[w \rightleftharpoons v]}$$

Let *R* and $R^{\prime }\mathop {=}\limits ^{\tiny def }R[w \rightleftharpoons v]$ be as in Figs. 10 and 11. According to [16], the clause label $C^{\prime }=\ell ^{\prime }(v)={\mathrm {Res}}(\ell ^{\prime }(w),\ell ^{\prime }(v_2),t_0)$ in Fig. 11 differs from $C=\ell (v)$ in Fig. 10 in the following two cases:

1. If $t_0 \in C_3$ then $t_0 \in C$, but $t_0 \notin C^{\prime }$.
2. If $t_1 \in C_2$ then $t_1 \notin C$, but $t_1 \in C^{\prime }$.

As explained in Remark 4, the former case does not occur in resolution chains generated by CDCL, since resolved literals are never reintroduced. In the second case, however, the swap *introduces* a literal into an (intermediate) resolvent. Since the resolution chain is regular, this literal propagates to the final resolvent of the chain, potentially invalidating the clausal proof.

Instead of prohibiting the transformation in general, however, it is possible to analyse the underlying resolution proof *R* to determine whether the literal introduced by the transformation is eliminated along all paths to the sink of the proof [3, 9, 19]. The set of literals eliminated along all paths from $v\in V_R$ to $\mathtt {s}_R$ can be defined as the *meet-over-all-paths* in the terminology of data-flow analysis:

#### **Definition 14**

(*Safe Literals*) Let $R=(V_R,E_R,\ell _R,\mathtt {s}_R)$ be a resolution refutation. The *safe literals* $\sigma (v)$ of a vertex $v\in V_R$ are defined inductively as follows:

$$\begin{aligned} \begin{aligned} {\mathrm {rlit}}(v,w)&= t~\text {s.t.}~t\in \ell (v),{\mathrm {var}}(t)={ piv }(w), \exists u\ne w\,\cdot \,(u,w)\in E_R\wedge {\mathrm {rlit}}(u,w)=\overline{t}\\ \sigma (v)&=\left\{ \begin{array}{ll} \emptyset &{} \quad \text {if}~v=\mathtt {s}_R\\ \bigcap _{(v,w)\in E_R} \left( \sigma (w) \cup \{{\mathrm {rlit}}(v,w)\}\right) &{} \quad \text {otherwise} \end{array}\right. \\ \end{aligned} \end{aligned}$$

A solution to the data-flow equation in Definition 14 can be computed in linear time since the graph *R* is acyclic. For the proof to the left of Fig. 9 we obtain $\sigma (v)=\{x_3\}$ and $\sigma (w)=\{\overline{x}_2,x_3\}$, for instance.

Let *v* be the final vertex of the trivial resolution derivation that corresponds to a given resolution chain. A swap of two vertices of the chain that introduces a literal *t* in $\ell (v)$ is admissible iff $t\in \sigma (v)$. Accordingly, the literal *t* is introduced in the conclusion (final resolvent, respectively) of the chain. The proof remains valid since *t* is subsequently eliminated.

#### *Example 4*

**Fig. 12:**
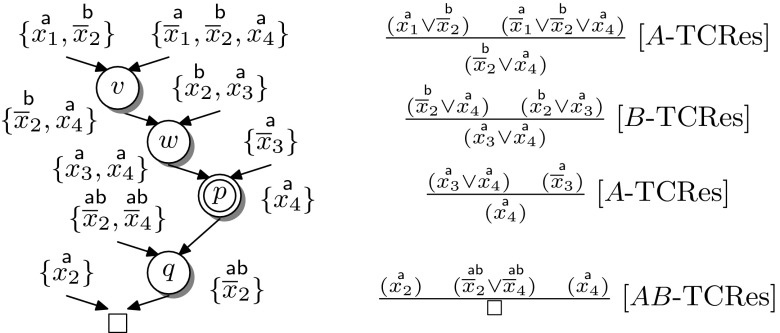
Two resolution chains and a corresponding clausal proof (after splitting)

Figure 12 shows a refutation with two chains generated by a CDCL solver, where the vertex *p* marks the end of the first chain. As in Example 3, the pivot order $\mathop {x_1}\limits ^{{\textsf {a}}}$, $\mathop {x_2}\limits ^{{\textsf {b}}}$, $\mathop {x_3}\limits ^{{\textsf {a}}}$ of the first chain enforces a split resulting in the TraceCheck resolution steps on the right side in Fig. 12. Similarly to the example in Fig. 9, reordering of the vertices *w* and *v* results in the introduction of the literal $\mathop {\overline{x}_2}\limits ^{{\textsf {b}}}$ in $\ell (p)$. The transformation is safe, however, since $\overline{x}_2\in \sigma (p)$. The transformation yields the following uniformly labelled TraceCheck resolution steps:


The interpolation system in Definition 13 remains applicable to the transformed clausal proof, since conclusions of TraceCheck resolution steps may always be weakened. The transformation may, however, affect the labelling of the pivots of the subsequent resolution steps. This might be undesirable, if it forces us to split subsequent chains. It is possible to avoid a change of the labelling by computing *safe labels* for the literals in a proof.

#### **Definition 15**

(*Safe Labels*) Given a refutation $R=(V_R,E_R,\ell _R,\mathtt {s}_R)$, the mapping $\varsigma : V_R\times \mathtt {Lit}\rightarrow {\mathcal {S}}$ (where  as in Definition 4) is defined inductively as follows:

2 $$\begin{aligned} \begin{aligned} {\mathrm {litlab}}(u,v,t)&= \left\{ \begin{array}{ll} L(v^+,{\mathrm {var}}(t)) \sqcup L(v^-,\overline{{\mathrm {var}}(t)}) &{} \quad \text {if}~t={\mathrm {rlit}}(u,v)\\ \varsigma (v, t) &{}\quad \text {otherwise}\\ \end{array} \right. \\ \varsigma (v,t)&=\left\{ \begin{array}{ll} \bot &{} \quad \text {if}~v=\mathtt {s}_R\\ \sqcap _{(v,w)\in E} {\mathrm {litlab}}(v,w, t) &{} \quad \text {otherwise} \end{array}\right. \\ \end{aligned} \end{aligned}$$

Given a vertex $v\in V_R$ and a literal $t\in \ell (v)$, we call $\varsigma (v,t)$ the safe label of *t*.

The safe labels $\varsigma$ are computed in lockstep with $\sigma$ (Definition 14). Whenever a literal $t\in \sigma (v)$ introduced into $\ell (v)$ is labelled such that $L(v,t)\sqsubseteq \varsigma (v,t)$, then the labelling of the pivots in the subsequent resolution steps remains unchanged [9].

#### *Example 5*

For the resolution refutation in Fig. 12 we obtain . Swapping the vertices *v* and *w* introduces $\overline{x}_2$ in $\ell (p)$ with $L(p,\overline{x}_2)={\textsf {a}}$. Consequently, the labelling of the pivot in the final resolution step is preserved.

The empirical evaluation in the following section motivates the use of interpolation systems for clausal proofs.

### Empirical Results

We implemented the labelled interpolation system for clausal proofs as an extension to the TraceCheck-tool.^2^ TraceCheck ’s original purpose is the verification of the output of SAT solvers, based on proof certificates stored in the TraceCheck-format.

Our interpolation system can be easily incorporated into TraceCheck. The only significant change arises from splitting the resolution chains to establish that ${\mathsf {Itp}}(L)$ is defined for a given labelling function *L*, as described in Sect. 4.1. Our implementation currently does not try to reduce the number of splits by means of reordering.

For the experimental evaluation of our implementation, we use benchmarks from reactive synthesis [8] obtained via the interpolation-based relation determinisation technique presented in [31]. We use PicoSAT 957 [5] to obtain clausal proofs in the TraceCheck-format. We limit the proofs to those with a file size between 100 kB and 10 MB, resulting in 133 benchmarks. We label the literals in *A*-clauses ${\textsf {a}}$ and the literals in *B*-clauses ${\textsf {b}}$, which provably results in the introduction of fewer literals than other labellings [9, 15]. All experiments were executed on an Intel Core i5 M560 at 2.67 GHz and with 8 GB of RAM.

**Fig. 13:**
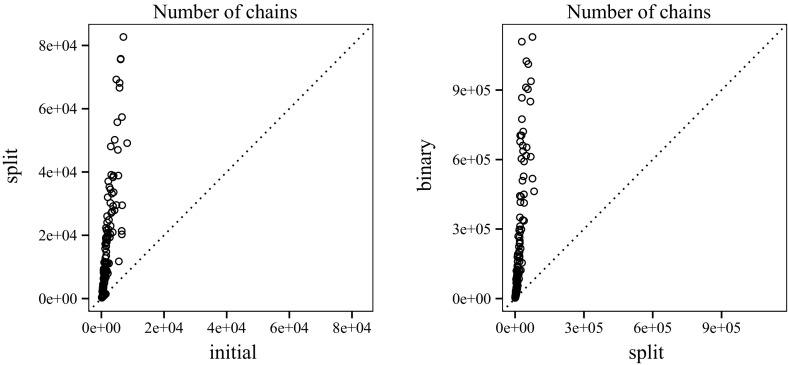
Number of chains before and after splitting; binary resolution steps

**Fig. 14:**
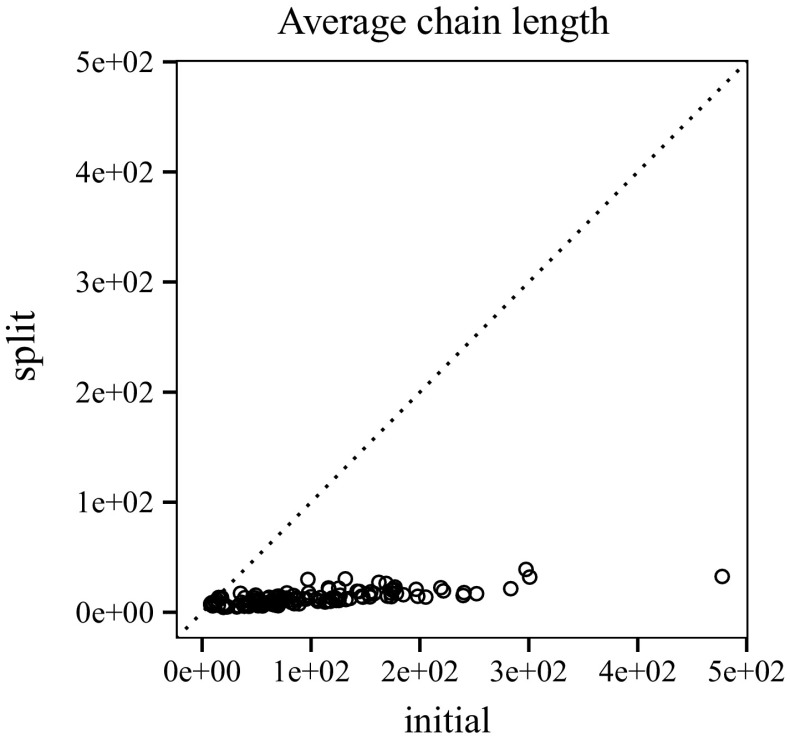
Average chain length

**Fig. 15:**
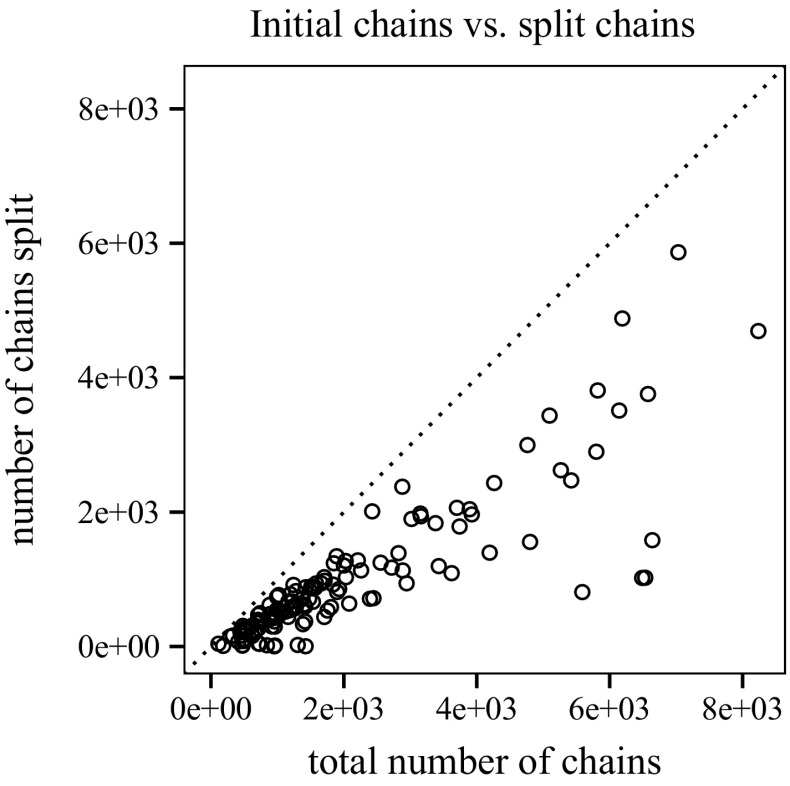
Chains that need to be split (46 %)

To measure the impact of transforming a clausal proof for labelled interpolation, we look at proofs before (initial) and after (split) splitting (Fig. 13). Using TraceCheck ’s -b option (binary), we also compare the clausal interpolation system to the conventional interpolation system for binary resolution proofs (presented in Sect. 2.2).^3^ Fig. 14 shows the average length of chains before and after splitting. On average, 44.86 % of the chains generated by TraceCheck need to be split to enable interpolation (Fig. 15).

**Fig. 16:**
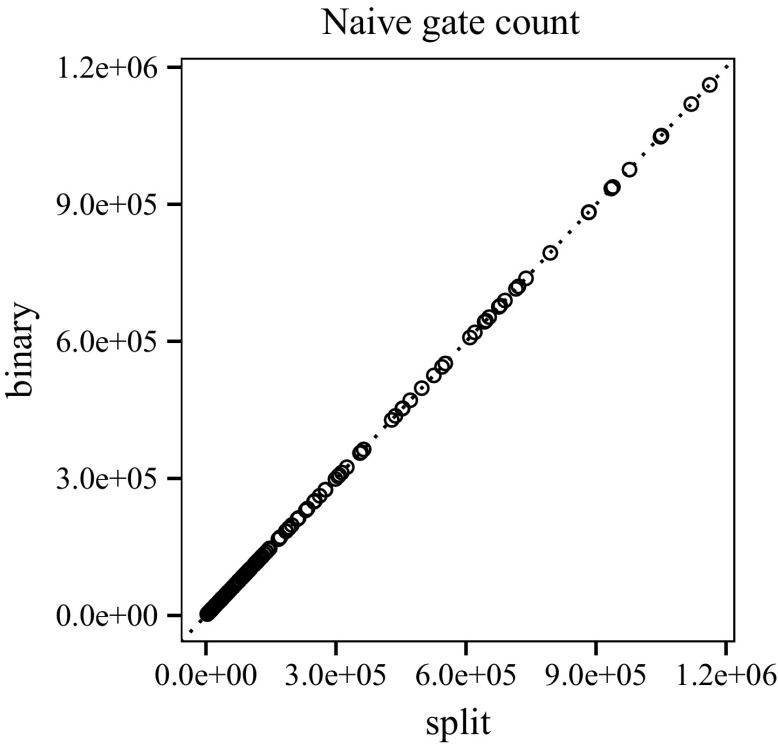
Number of gates before reduction

**Fig. 17:**
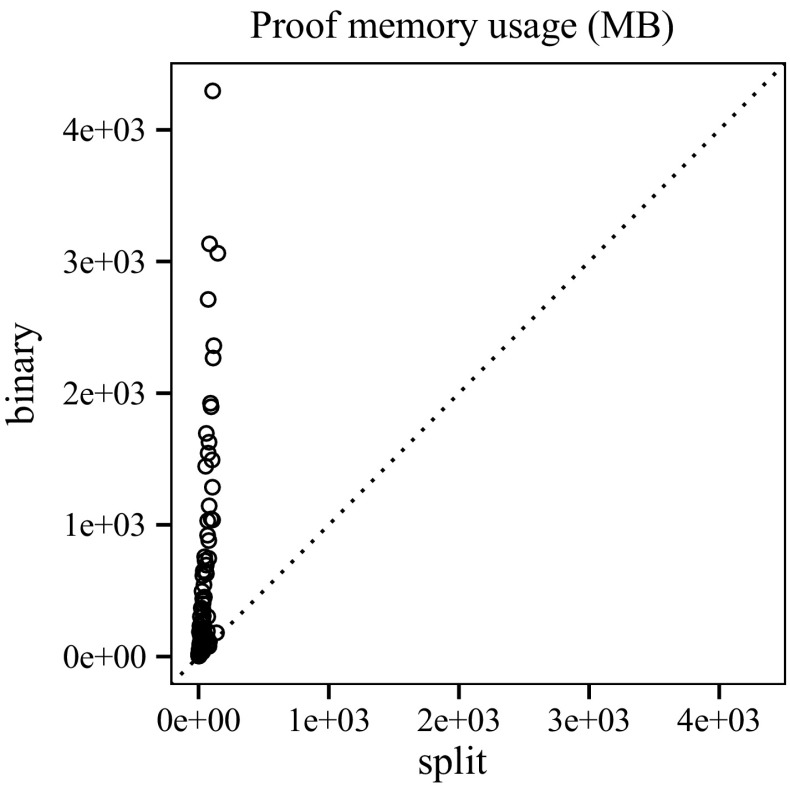
Memory usage

Figure 16 compares the number of Boolean operations in the interpolants generated by clausal interpolation and binary interpolation. The difference is negligible, since *n*-ary conjunctions are encoded by binary gates. Figure 17 shows the memory consumption of our interpolation systems (in megabytes). The plot for run-time has a similar shape. The average run-time for split proofs is 0.9 s and 5.49 s for binary proofs. The quantiles are as follows:

**Table:** 

	0 %	25 %	50 %	75 %	100 %
split (s)	0.01	0.17	0.52	1.40	4.85
binary (s)	0.06	0.63	1.79	5.55	54.09

**Fig. 18:**
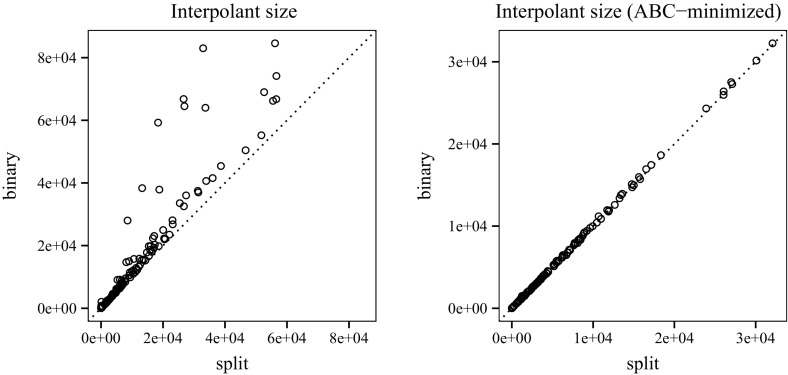
Size of AIG circuit; before and after reduction by ABC

We use the And-Inverter-Graph (AIG) library AIGER^4^ to store interpolants. The library performs trivial simplifications and structural hashing to keep the circuit size small. The graph on the left of Fig. 18 shows that the interpolants extracted from clausal proofs are consistently smaller than interpolants generated by the conventional interpolation technique.

Finally, we use ABC [12] to gather statistics about the interpolant and to reduce the circuit size further with the following commands: strash; balance; fraig; refactor -z; rewrite -z; fraig;. After reduction, the sizes of the interpolants extracted from clausal proofs and from binary proofs are similar. We emphasise that interpolation based on clausal proofs is superior with respect to memory consumption and the intermediate size of interpolants.

## Local Refutations and Hyper-Resolution

Jhala and McMillan demonstrate in [30, Theorem 3] that the applicability of propositional interpolation systems is not restricted to propositional logic. If a first-order refutation *R* has a certain structure, namely if for each inference step in *R* the antecedents as well as the conclusion are either entirely in ${\mathcal {L}}({A})$ or in ${\mathcal {L}}({B})$, then one can use a propositional interpolation system (such as the ones in Sects. 2.2 and 3) to construct an interpolant that is a Boolean combination of the formulae in *R*. Kovács and Voronkov subsequently arrived at a similar result [32].

We recapitulate the results from [30, 32] before we proceed to show that our interpolation system from Definition 9 generalises the system of [32] as well as a variation of [32] presented in [55].

### **Definition 16**

(*Local Refutation*) An (*A*, *B*)-refutation *R* in a given inference system for first-order logic is *local* if there exists a total *partitioning* function $\pi _R: V_R\rightarrow \{A,B\}$ such that for all edges $(v_1,v_2)\in E_R$ we have $\ell _R(v_1),\ell _R(v_2)\in {\mathcal {L}}({\pi _R(v_2)})$.

While proofs in general do *not* have this property, there is a variety of decision procedures that yield local (ground) refutations. The construction of local proofs is addressed in [20, 30, 32, 41], to name only a few.

The following operation, which resembles the constructions in [32, Lemma 8], [30, Theorem 3], and [20, Section 5.5]), extracts a premise in ${\mathcal {L}}({A})$ (${\mathcal {L}}({B})$, respectively) for a vertex $v\in V_R$ with $\pi (v)=A$ ($\pi (v)=B$, respectively) from a local refutation *R*.

### **Definition 17**

(*A*-*Premise*, *B*-*Premise* Let *R* be a local (*A*, *B*)-refutation with partitioning function $\pi$, and let $v\in V_R$ such that $\pi (v)=A$. Then

$$\begin{aligned} \begin{aligned} A&\text {-premise}\,(v)\mathop {=}\limits ^{\tiny def }\\&\{u\,\vert \, (u,v)\in E_R~\text {and}~\pi (u)=B~\text {or}~u~\text {is initial}\,\}\;\cup \\&\bigcup \{A\text {-premise}\,(u)\,\vert \, (u,v)\in E_R~\text {and}~\pi (u)=A\,\}. \end{aligned} \end{aligned}$$

*B*-premise(*v*) is defined analogously.

Intuitively, *A*-premise(*v*) comprises the leaves of the largest sub-derivation *S* rooted at *v* such that $\pi (u)=A$ for all internal vertices $u\in V_S$.^5^ If the underlying inference system is sound, we have $\{\ell (u)\,\vert \, u\in A\text {-premise}(v)\}\models \ell (v)$. If, moreover, $\ell (v)$ as well as all formulae of *A*-premise(*v*) are *closed*, we make the following observation (c.f. related results in [32, Lemma 1] and [20, Lemma 3]):

### **Corollary 1**

Let *R* be a local closed refutation in a sound inference system, and let $v\in V_R$ an internal vertex such that $\pi _R(v)=A$. Then, the following Horn clause is a tautology:

3 $$\begin{aligned} \bigvee _{u\in A\text {-premise}(v)}\lnot \ell _R(u)\vee \ell _R(v) \end{aligned}$$

A similar claim holds for the case in which $\pi (v)=B$.

Corollary 1 is a pivotal element in our proof of the following theorem:

### **Theorem 5**

(c.f. [30, Theorem 3]) Let *R* be a closed local (*A*, *B*)-refutation in a sound inference system. Then one can extract a Craig interpolant from *R* using a propositional interpolation system.

### *Proof*

Let $v\in V_R$ be such that $\pi (v)=A$. If *v* is initial, then either *A* or *B* contains the unit clause $C_v=\ell (v)$. Otherwise, according to Corollary 1, the clause $C_v=(\{\lnot \ell (u)\,\vert \,u\in A\text {-premise}(v)\}\vee \ell (v))$ is tautological (and therefore implied by *A*). Moreover, it follows from Definition 16 that if $u\in A$-premise(*v*) is not an initial vertex of *R* then $\ell _R(u)\in {\mathcal {L}}({A})\cap {\mathcal {L}}({B})$ holds. Accordingly, $C_v\in {\mathcal {L}}({A})$, and we add $C_v$ to *A*. A similar argument holds for $v\in V_R$ with $\pi (v)=B$.

By construction, the resulting set of clauses $C_v$, $v\in V_R$, is propositionally unsatisfiable [30, 32]; also, each clause is implied by either *A* or *B*. Moreover, all literals with $t\in {\mathcal {L}}({A}){\setminus }{\mathcal {L}}({B})$ ($t\in {\mathcal {L}}({B}){\setminus }{\mathcal {L}}({A})$, respectively) are local to *A* (*B*, respectively). Accordingly, it is possible to construct an interpolant for (*A*, *B*) using the interpolation systems presented in Sects. 2.2 and 3. $\square$

### *Example 6*

**Fig. 19:**
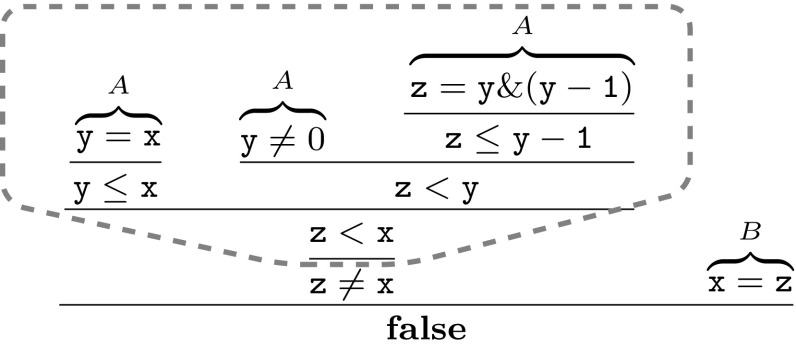
Refutation of $(\mathtt{y}=\mathtt{x})\wedge (\mathtt{y}\ne 0)\wedge (\mathtt{z}=\mathtt{y} \& \mathtt{(y-1)})\;\wedge \;(\mathtt{x}=\mathtt{z})$; A-premise of $\mathtt{z}<\mathtt{x}$

Figure 19 shows an (*A*, *B*)-refutation for $A\equiv (\mathtt{y}=\mathtt{x})\wedge (\mathtt{y}\ne 0)\wedge (\mathtt{z}=\mathtt{y} \& \mathtt{(y-1)})$ and $B\equiv (\mathtt{x}=\mathtt{z})$, where $\mathtt{x}, \mathtt{y}, \mathtt{z}$ are bit-vectors and & denotes bit-wise conjunction. Let vertex *v* be such that $\ell (v)=(\mathtt{z}<\mathtt{x})$ and $\pi (v)=A$. The dashed line in Fig. 19 indicates the sub-proof rooted at *v*, whose leaves constitute the *A*-premise of *v*. Following the construction in the proof of Theorem 5, we obtain the following hyper-resolution step with conclusion $\ell (v)$.

$$ \begin{aligned} \frac{(\mathtt{y}=\mathtt{x})\quad (\mathtt{y}\ne 0)\quad (\mathtt{z}=\mathtt{y} \& \mathtt{(y-1)})\quad \overbrace{\left( \overline{(\mathtt{y}=\mathtt{x})}\vee \overline{(\mathtt{y}\ne 0)}\vee \overline{(\mathtt{z}=\mathtt{y} \& \mathtt{(y-1)})}\vee (\mathtt{z}{<}{} \mathtt{x})\right) }^{\text {tautology in}\, {\mathcal {L}}(A)}}{\mathtt{z}{<}{} \mathtt{x}} \end{aligned}$$

Consider the vertex *w* with $\ell (w)=(\mathtt{z}\ne \mathtt{x})$ and $\pi (w)=B$. The corresponding *B*-premise is $\{v\}$, resulting in the resolution step ${\mathrm {Res}}(\{(\mathtt{z}<\mathtt{x})\},\{\lnot (\mathtt{z}<\mathtt{x}),(\mathtt{z}\ne \mathtt{x})\},(\mathtt{z}<\mathtt{x}))$ with conclusion $(\mathtt{z}\ne \mathtt{x})$.

Kovács and Voronkov avoid the explicit construction of a resolution proof by defining their interpolation system directly on the local proof [32, Theorem 11]:

### **Definition 18**

**Fig. 20:**
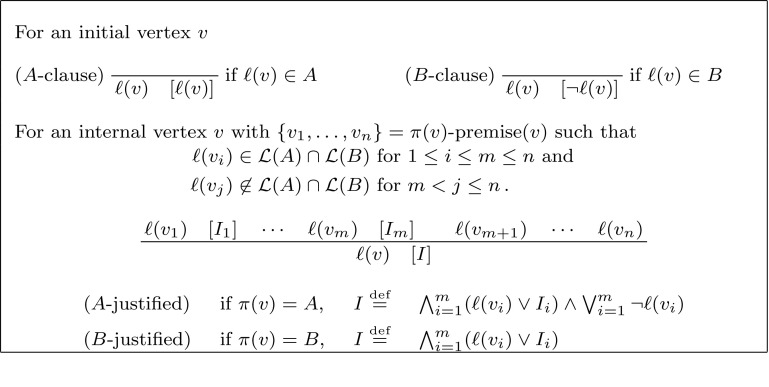
Interpolation system ${\mathsf {Itp}}_{KV}$ for local proofs

Let *R* be a local and closed (*A*, *B*)-refutation. The interpolation system ${\mathsf {Itp}}_{KV}$ maps vertices $v\in V_R$, for which $\ell _R(v)\in {\mathcal {L}}({A})\cap {\mathcal {L}}({B})$ holds, to partial interpolants as defined in Fig. 20.

### *Remark*

In addition to the condition in Definition 16, Kovács and Voronkov require that for each $v\in V_R$ with predecessors $v_1, \ldots , v_n$, $\ell (v)\in {\mathcal {L}}({A})\cap {\mathcal {L}}({B})$ if $\ell (v_i)\in {\mathcal {L}}({A})\cap {\mathcal {L}}({B})$ for all $i\in \{1, \ldots , n\}$. A local derivation satisfying this condition is *symbol-eliminating*, i.e., it does not introduce “irrelevant” symbols. This technical detail allows the leaves of *R* to be merely implied by *A* (or *B*) instead of being actual elements of *A* (*B*, respectively), while preserving the correctness of the interpolation system. This effectively enables interpolation for *non-closed* formulae (*A*, *B*).

We proceed to show one of the main results of this paper, namely that our interpolation system ${\mathsf {Itp}}$ from Definition 9 is able to simulate the interpolation system ${\mathsf {Itp}}_{KV}$.

### **Theorem 6**

Let *R* be a local and closed (*A*, *B*)-refutation. Then we can construct a hyper-resolution refutation *H* of (*A*, *B*) and a locality preserving labelling function *L* such that for each $v\in V_R$ with $\ell _R(v)\in {\mathcal {L}}({A})\cap {\mathcal {L}}({B})$ there exists a corresponding vertex $u\in V_H$ such that ${\mathsf {Itp}}_{KV}(R) (v)\Leftrightarrow {\mathsf {Itp}}_1(L,H) (u)$.

### *Proof sketch*

We demonstrate that it is possible to construct a hyper-resolution refutation *H* of (*A*, *B*) in which each internal step of ${\mathsf {Itp}}_{KV}$ is simulated using *two* hyper-resolution steps. The induction hypothesis is that for each internal vertex $v\in V_R$ with $\{v_1, \ldots , v_n\}=\pi (v)\text {-premise}(v)$ and *m* as in Definition 18, we have vertices $\{u_1, \ldots , u_n\}\subseteq V_H$ such that

1. $$\ell _H(u_i)=\ell _R(v_i)$$ for $$1\le i\le n$$, and
2. $${\mathsf {Itp}}_1(L,H) (u_i)\Leftrightarrow {\mathsf {Itp}}_{KV}(R) (v_i)$$ for $$1\le i \le m$$, and
3. $${\mathsf {Itp}}_1(L,H) (u_j)=\left\{ \begin{array}{ll} {\mathsf {F}} &{} \text {if }\ell (v_j)\in A\\ {\mathsf {T}} &{} \text {if }\ell (v_j)\in B \end{array}\right. $$ for $$m< j\le n$$.

We add an auxiliary vertex labelled with the clause $\lnot \ell _H(u_1)\vee \cdots \vee \lnot \ell _H(u_n)\vee \ell _R(v)$, which, by Corollary 1 and by Definition 16, can be regarded as element of formula $\pi (v)$ (see proof of Theorem 5). The first hyper-resolution step eliminates the literals local to $\pi (v)$; the interpolants and labels are indicated for $\pi (v)=A$:The second hyper-resolution step eliminates the shared literals $\ell _H(u_i)$ (for $1\le i \le m$). Again, the labels and interpolants are for the case that $\pi (v)=A$:The sink of this resolution step is the vertex $u\in V_H$ such that $\ell _H(u)=\ell _R(v)$ and ${\mathsf {Itp}}_1(L,H) (u)={\mathsf {Itp}}_{KV}(v)$. $\square$

We proceed to show that our system for hyper-resolution also generalises another existing interpolation system for local refutations. In [55], we introduced the following variation of the interpolation system in Definition 18:

### **Definition 19**

Let ${\mathsf {Itp}}_{W}$ be the interpolation system as described in Definition 18, except for the following modification: 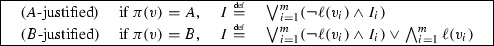


The following theorem states that the interpolation system in Definition 9 is powerful enough to simulate ${\mathsf {Itp}}_{W}$.

### **Theorem 7**

Let *R* be a local and closed (*A*, *B*)-refutation. Then we can construct a hyper-resolution refutation *H* of (*A*, *B*) and a locality preserving labelling function *L* such that for each $v\in V_R$ with $\ell _R(v)\in {\mathcal {L}}({A})\cap {\mathcal {L}}({B})$ there exists a corresponding vertex $u\in V_H$ such that ${\mathsf {Itp}}_{W}(R) (v)\Leftrightarrow {\mathsf {Itp}}_2(L,H) (u)$.

The proof is essentially equivalent to the proof of Theorem 6. Moreover, as a consequence of Theorem 2, ${\mathsf {Itp}}_{KV}$ is *stronger* than ${\mathsf {Itp}}_{W}$.

### **Corollary 2**

Let *R* be a closed local (*A*, *B*)-refutation in a sound inference system. Then ${\mathsf {Itp}}_{KV}(R)\Rightarrow {\mathsf {Itp}}_{W}(R)$.

## Related Work

There is a vastly growing number of different interpolation techniques; a recent survey of interpolation in decision procedures is provided by [10]. An exposition of interpolation techniques for SMT solvers can be found in [13]. The work of Yorsh and Musuvathi [58] enables the combination of theory-specific and propositional interpolation techniques [16, 28, 33, 39, 42].

The novel interpolation system presented in Sect. 3 extends our prior work on propositional interpolation systems [16]. The idea of using labelling functions (initially introduced in [50] in the context of LTL vacuity detection to determine the *peripherality* of variables in resolution proofs) is common to both approaches. In [16], the partial interpolants are determined by the labelling of the literals in the initial vertices, while the system presented in Sect. 3 adds an additional degree of freedom by allowing us to make a choice at each internal node.

Recent work by Vizel and Gurfinkel [24] addresses the construction of interpolants from clausal/DRUP proofs (whose size is reduced by means of trimming [25]). Their interpolation system splits partial interpolants into two components, one of which is kept in CNF. Their algorithm restructures the DRUP proof on-the-fly in order to increase the size of the component kept in CNF. Earlier work by Vizel et al. [53] targets the construction of interpolants in CNF by first constructing an over-approximation of an interpolant, which is then refined using inductive strengthening [11].

There is a number of techniques to reduce the size of resolution proofs [3, 9, 19]. These techniques target binary resolution proofs, however. The combination of labelled interpolation systems for binary resolution proofs and proof reduction has also been studied extensively by Rollini et al. [44, 45].

A number of interpolation techniques rely on local proofs (e.g., [20, 30, 32, 36, 41]). Not all interpolation techniques are based on local proofs, though: McMillan’s interpolating inference system for equality logic with uninterpreted functions and linear arithmetic [40], for instance, performs an implicit conversion of the proof. In [35], propositional proofs of bit-vector formulas are lifted to proofs in equality logic. The approach presented in [47] avoids the construction of proofs altogether and handles theory combination by reduction to a base theory as in [51] or [52]. InterHorn [23] extracts interpolants from first-order resolution proofs generated by a Horn-clause solver. Sharma et al. show how to compute interpolants without proofs using machine learning techniques [48].

Hoder et al. [26] present a technique that enables the variation of interpolants by fine-tuning the partitioning in Definition 16. In Example 6, for instance, changing $\pi (w)=B$ to $\pi (w)=A$ results in propositional proof that does not contain the literal $(\mathtt{z} < \mathtt{x})$. Accordingly, the term does not occur in the resulting interpolant. This approach can be combined with our interpolation system in a straight forward manner.

An extension of [16] to sequence interpolants is presented in [46]. A survey of interpolation-based model checking techniques is provided in [54]. Interpolation-based synthesis is discussed in [27, 31]. Other applications of interpolation algorithms include fault localization [59] and error explanation [18, 57], where the quality of interpolants can impact the utility of the diagnosis.

## Consequences and Conclusion

We present a novel interpolation system for hyper-resolution proofs which generalises our previous work [16]. We subsequently generalise this interpolation system to clausal proofs, generated by contemporary SAT solvers. By defining a rule that addresses hyper-resolution or clausal resolution steps (introduced by pre-processing [21] or extracted from resolution chains), we avoid the construction of intermediate partial interpolants, resulting in reduced memory consumption and smaller intermediate interpolants. As future work, we will investigate whether proof restructuring [24] and heuristics based on proof analysis [9] can result in a further reduction of splitting.

By applying our technique to local proofs, we combine a number of first-order [32, 55] and propositional interpolation techniques [28, 33, 39, 42] into one *uniform* interpolation approach. As in [30], our approach avoids an explicit theory combination step [58]. Therefore, it enables the variation of interpolant strength and the elimination of non-essential literals across the theory boundary.

## Appendix 1: Proofs

### *Remark*

The *downward projection* of a clause $\ell (v)=C$ at vertex *v* with respect to ${\textsf {c}} \in {\mathcal {S}}$ is defined as $\ell (v)\downharpoonright _{{\textsf {c}},L} \mathop {=}\limits ^{\tiny def }\{t \in \ell (v)\,\vert \, L(v,t) \sqsubseteq {\textsf {c}} \}$, the respective *upward projection* is $\ell (v)\upharpoonright _{{\textsf {c}},L} \mathop {=}\limits ^{\tiny def }\{t \in \ell (v)\,\vert \, {\textsf {c}} \sqsubseteq L(v,t)\}$ (cf. Sect. 2.2). It follows from condition 1 in Definition 4 that $\ell (v)\downharpoonright _{\bot ,L}=\emptyset$ for all vertices *v*. Therefore, the following two equalities hold for any clause $C=\ell (v)$ in a refutation *R*:

- $$C\downharpoonright _{{\textsf {b}},L}=(C\upharpoonright _{{\textsf {b}},L}{\setminus }C\upharpoonright _{{\textsf {a}},L})$$
- $$C\downharpoonright _{{\textsf {a}},L}=(C\upharpoonright _{{\textsf {a}},L}{\setminus }C\upharpoonright _{{\textsf {b}},L})$$

We make repeated use of these equalities in this section. Moreover, our proofs use the following propositions:

### **Proposition 1**

The implication

$$\begin{aligned} \left( \bigwedge _{i=1}^n (x_i \vee I_i) \wedge \left( I_{n+1}\vee \bigvee _{i=1}^n\overline{x}_i \right) \right) \Rightarrow \left( \bigvee _{i=1}^n (\overline{x}_i \wedge I_i) \vee \left( I_{n+1}\wedge \bigwedge _{i=1}^n x_i \right) \right) \end{aligned}$$

is a tautology.

### *Proof*

This follows from the fact that the conjunction

$$\begin{aligned} \left( \bigwedge _{i=1}^n (x_i \vee I_i) \wedge \left( I_{n+1}\vee \bigvee _{i=1}^n\overline{x}_i \right) \right) \wedge \left( \bigwedge _{i=1}^n (x_i \vee \lnot I_i) \wedge \left( \lnot I_{n+1}\vee \bigvee _{i=1}^n \overline{x}_i \right) \right) \end{aligned}$$

is unsatisfiable (by hyper-resolution). Note that the implication in the other direction does not hold; a simple counterexample for the case $n=2$ is the assignment $x_1=x_2={\mathsf {F}}$, $I_1={\mathsf {T}}$, and $I_2=I_3={\mathsf {F}}$. $\square$

### **Proposition 2**

Let $\bigwedge _{i=1}^n P_i$ be unsatisfiable. Then the implication

$$\begin{aligned} \left( \bigwedge _{i=1}^n (I_i \vee P_i) \right) \Rightarrow \left( \bigvee _{i=1}^n (I_i \wedge \lnot P_i) \right) \end{aligned}$$

is a tautology.

### *Proof*

This follows from the fact that

$$\begin{aligned} \bigwedge _{i=1}^n (I_i \vee P_i) \wedge \bigwedge _{i=1}^n (\lnot I_i \vee P_i)\quad \equiv \quad \bigwedge _{i=1}^n (I_i \vee P_i) \wedge (\lnot I_i \vee P_i) \end{aligned}$$

is unsatisfiable [since ${\mathrm {Res}}((I_i \vee P_i), (\lnot I_i \vee P_i), I_i) = P_i$ and $\bigwedge _{i=1}^n P_i$ is unsatisfiable]. $\square$

### **Theorem 1**

(Correctness) For any (*A*, *B*)-refutation *R* (where *R* is a hyper-resolution proof) and locality preserving labelling function *L*, ${\mathsf {Itp}}(L,R)$ (if defined) is an interpolant for (*A*, *B*).

### *Proof*

By induction over the structure of the (*A*, *B*)-refutation *R*. Let *I* be the partial interpolant at a vertex *v* labelled with a clause $C = \ell (v)$. We show that every such *I* and *C* satisfy the following conditions:

1. $$A\wedge \lnot (C\upharpoonright _{{\textsf {a}},L})\Rightarrow I$$,
2. $$B\wedge \lnot (C\upharpoonright _{{\textsf {b}},L})\Rightarrow \lnot I$$, and
3. $$\text {Var}(I)\subseteq \text {Var}(A)\cap \text {Var}(B)$$.

For the sink *v* with $\ell (v)=\Box$, this establishes Theorem 1. The labelling function *L*, being unique in this proof, is omitted from subscripts.

*Base case* Let *v* be an initial vertex and let $C=\ell _R(v)$.

1. $$C\in A$$:
  - $A\wedge \lnot (C\upharpoonright _{{\textsf {a}}})\Rightarrow C\downharpoonright _{{\textsf {b}}}$, is equivalent to $A\Rightarrow (C\upharpoonright _{{\textsf {b}}}{\setminus }C\upharpoonright _{{\textsf {a}}}{L})\vee C\upharpoonright _{{\textsf {a}}}$. This holds because $(C\upharpoonright _{{\textsf {b}}}{\setminus }C\upharpoonright _{{\textsf {a}}})\vee C\upharpoonright _{{\textsf {a}}}=C$, and $A\Rightarrow C$ because $C\in A$.
  - $B\wedge \lnot (C\upharpoonright _{{\textsf {b}}})\Rightarrow \lnot (C\downharpoonright _{{\textsf {b}}})$, is equivalent to $B\wedge (C\upharpoonright _{{\textsf {b}}}{\setminus } C\upharpoonright _{{\textsf {a}}}) \Rightarrow C\upharpoonright _{{\textsf {b}}}$. This holds because $(C\upharpoonright _{{\textsf {b}}}{\setminus } C\upharpoonright _{{\textsf {a}}})\subseteq C\upharpoonright _{{\textsf {b}}}$ and clauses represent disjunctions.
  - For all literals $t\in (C\upharpoonright _{{\textsf {b}}}{\setminus } C\upharpoonright _{{\textsf {a}}})$ the following conditions hold:
    - $${\mathrm {var}}(t)\in {\mathrm {Var}}(A)$$, since $$C\in A$$.
    - $L(v,t)={\textsf {b}}$. Therefore, by Definition 5, ${\mathrm {Var}}(t)\in {\mathrm {Var}}(B)$.
    This establishes that ${\mathrm {Var}}(C\upharpoonright _{{\textsf {b}}}{\setminus } C\upharpoonright _{{\textsf {a}}})\subseteq {\mathrm {Var}}(A)\cap {\mathrm {Var}}(B)$.
2. $$C\in B$$: Symmetric to $$C\in A$$.

*Induction step* We first prove a useful equality. Let *v* be an internal vertex of *R* with ancestors $\{v^+_1, \ldots , v^+_n, v^-\}$. We claim that

4 $$\begin{aligned} \bigvee _{i=1}^n (\ell (v^+_i) {\setminus }\{x_i\}) \upharpoonright _{{\textsf {c}}}\, \vee \, (\ell (v^-) {\setminus } \{\overline{x}_1, \ldots , \overline{x}_n \}) \upharpoonright _{{\textsf {c}}} = \ell (v)\upharpoonright _{{\textsf {c}}} \end{aligned}$$

holds for a symbol ${\textsf {c}} \in \{{\textsf {a}}, {\textsf {b}} \}$. This is because for any $t \in \ell (v^+_i)$ (for $1\le i\le n$), if ${\mathrm {var}}(t) \ne x_i$, then $L(v^+_i,t) \sqsubseteq L(v,t)$. The same holds for $t \in \ell (v^-)$. Thus, if ${\mathrm {var}}(t) \ne x_i$ and $t \in \ell (u)\upharpoonright _{{\textsf {c}}}$ for $u\in \{v^+_1, \ldots , v^+_n,v^-\}$, then $t \in \ell (v) \upharpoonright _{{\textsf {c}}}$. Conversely, if $t \in \ell (v)\upharpoonright _{{\textsf {c}}}$, then ${\textsf {c}} \sqsubseteq L(v,t)$ by the definition of projection. From Definition 5, $L(v,t)=L(v^+_1,t)\sqcup \cdots \sqcup L(v^+_n,t)\sqcup L(v^-,t)$, thus, if ${\textsf {c}} \sqsubseteq L(v,t)$ and , then $\exists i\,\cdot \,{\textsf {c}} \sqsubseteq L(v^+_i,t)$ or ${\textsf {c}} \sqsubseteq L(v^-,t)$. It follows that $\exists i\,\cdot \,t \in (\ell (v^+_i){\setminus }\{x_i\}) \upharpoonright _{{\textsf {c}}}$ or $t \in ( \ell (v^-){\setminus } \{\overline{x}_1, \ldots , \overline{x}_n \}) \upharpoonright _{{\textsf {c}}}$.

For the induction step, let $\ell (v^+_i) = (x_i \vee C_i)$ and $\ell (v^-) = (\overline{x}_1\vee \cdots \vee \overline{x}_n \vee D)$. Due to the requirement that ${\mathsf {Itp}}(L,R)$ is defined, we may assume that $\forall i\in \{1, \ldots , n\}\,\cdot \,L(v^+_i, x_i)\sqcup L(v^-, \overline{x}_i)={\textsf {c}}$ (for a fixed ${\textsf {c}}$) and perform a case split on ${\textsf {c}}$:

1. $$\forall i\in \{1, \ldots , n\}\,\cdot \,L(v^+_i, x_i)\sqcup L(v^-, \overline{x}_i)={\textsf {a}} $$: *Induction hypothesis*
  It follows that $A\wedge \lnot (C_i\upharpoonright _{{\textsf {a}}})\Rightarrow (x_i\vee I_i)$ for $i\in \{1, \ldots , n\}$ and $A\wedge \lnot (D\upharpoonright _{{\textsf {a}}}) \Rightarrow (I_{n+1}\vee \bigvee _{i=1}^n \overline{x}_i)$, and therefore
  $$\begin{aligned} A\wedge \underbrace{\bigwedge _{i=1}^n\lnot (C_i\upharpoonright _{{\textsf {a}}})\wedge \lnot (D\upharpoonright _{{\textsf {a}}})}_{\lnot (\bigvee _{i=1}^n C_i\vee D)\upharpoonright _{{\textsf {a}}},\text { by }(4)} \Rightarrow \bigwedge _{i=1}^n (x\vee I_i)\wedge (I_{n+1}\vee \bigvee _{i=1}^n \overline{x}_i). \end{aligned}$$
  By applying hyper-resolution on the right-hand side of the implication we conclude that
  $$\begin{aligned} A\wedge \bigwedge _{i=1}^n\lnot (C_i\upharpoonright _{{\textsf {a}}})\wedge \lnot (D\upharpoonright _{{\textsf {a}}}) \Rightarrow \bigvee _{i=1}^{n+1} I_i. \end{aligned}$$
  Similarly, we derive from the induction hypothesis that
  $$\begin{aligned} A\wedge \lnot (\bigvee _{i=1}^n C_i\vee D)\upharpoonright _{{\textsf {a}}} \Rightarrow \lnot I_{n+1}\wedge \bigwedge _{i=1}^n \lnot I_i, \end{aligned}$$
  and thus
  $$\begin{aligned} A\wedge \lnot (\bigvee _{i=1}^n C_i\vee D)\upharpoonright _{{\textsf {a}}} \Rightarrow \lnot \bigvee _{i=1}^{n+1} I_i. \end{aligned}$$
  $\text {Var}(\bigvee _{i=1}^{n+1}I_i) \subseteq \text {Var}(A)\cap \text {Var}(B)$ holds because ${\mathrm {Var}}(I_i)\subseteq {\mathrm {Var}}(A)\cap \text {Var}(B)$ for all $i\in \{1, \ldots , n\}$.
2. $\forall i\in \{1, \ldots , n\}\,\cdot \,L(v^+_i, x_i)\sqcup L(v^-, \overline{x}_i)={\textsf {b}}$: The proof is symmetric to the first case.
3. : *Induction hypothesis* Let $L(v^+_i,x_i)={\textsf {a}}$ and $L(v^-,\overline{x}_i)={\textsf {b}}$, $1\le i \le n$, for instance. We obtain the following induction hypothesis:
  In general, for an arbitrary labelling function *L*, this can always be extended to the induction hypothesis for , $1\le i \le n$:
  From this, it follows immediately that
  $$\begin{aligned} A\wedge \bigwedge _{i=1}^n \lnot (C_i\upharpoonright _{{\textsf {a}}})\wedge \lnot (D\upharpoonright _{{\textsf {a}}}) \Rightarrow \bigwedge _{i=1}^n(x_i\vee I_i) \wedge (\overline{x}_1\vee \cdots \vee \overline{x}_n\vee I_{n+1}), \end{aligned}$$
  and by applying the equality (4) we conclude that
  $$\begin{aligned} A\wedge \lnot (\bigvee _{i=1}^n C_i \vee D)\upharpoonright _{{\textsf {a}}} \Rightarrow \bigwedge _{i=1}^n(x_i\vee I_i) \wedge (I_{n+1}\vee \bigvee _{i=1}^n\overline{x}_i) \end{aligned}$$
  holds. This establishes the first condition for case 1 of $AB$ -HyRes; case 2 is covered by applying Proposition 1. Similarly, we derive
  $$\begin{aligned} B\wedge \lnot (\bigvee _{i=1}^n C_i\vee D)\upharpoonright _{{\textsf {b}}} \Rightarrow \bigwedge _{i=1}^n(x_i\vee \lnot I_i) \wedge (\overline{x}_1\vee \cdots \vee \overline{x}_n\vee \lnot I_{n+1}). \end{aligned}$$
  Note that this already establishes condition 2 for case 2 of $AB$-HyRes. By repeated application of resolution, one can further show that the right-hand side of this implication is inconsistent with $\bigwedge _{i=1}^n(x_i\vee I_i) \wedge (\overline{x}_1\vee \cdots \vee \overline{x}_n\vee I_{n+1})$. It follows that
  $$\begin{aligned} B\wedge \lnot (\bigvee _{i=1}^n C_i\vee D)\upharpoonright _{{\textsf {b}}} \Rightarrow \lnot \left( \bigwedge _{i=1}^n(x_i\vee I_i) \wedge (I_{n+1}\vee \bigvee _{i=1}^n\overline{x}_i)\right) , \end{aligned}$$
  which covers case 1 of $AB$-HyRes. Note that $x_i\in \text {Var}(A)\cap \text {Var}(B)$ due to  (for $1\le i \le n$) and Definition 5, and therefore ${\mathrm {Var}}(\bigwedge _{i=1}^n(x_i\vee I_i) \wedge (I_{n+1}\vee \bigvee _{i=1}^n\overline{x}_i)) \subseteq {\mathrm {Var}}(A)\cap {\mathrm {Var}}(B)$ holds.

$$\square $$

### **Lemma 1**

Let *L* and $L^{\prime }$ be labelling functions for an (*A*, *B*)-refutation *R*. If $L(v,t) \preceq L^{\prime }(v,t)$ holds for all initial vertices *v* and literals $t \in \ell (v)$, then $L \preceq L^{\prime }$.

### *Proof*

We show that $L(v,t)\preceq L^{\prime }(v,t)$ for all *v* in *R* by structural induction.

*Base case* If *v* in *R* is an initial vertex, $L(v,t)\preceq L^{\prime }(v,t)$ holds by assumption.

*Induction hypothesis* For an internal vertex *v* and literal *t*:

*Induction step* Let *v* be an internal vertex in *R* with ancestors $\{v^+_1, \ldots , v^+_n,v^-\}$, and let $\ell _R(v^+_i)=C_i\vee x_i$ and $\ell _R(v^-)=D\vee \overline{x}_1\vee \cdots \vee \overline{x}_n$.

We consider two cases:

1. If $$t \notin \ell (v)$$, then $$L(v,t) = L^{\prime }(v,t) = \bot $$.
2. If $t\in \ell (v)$, there are three cases:
  - If $L(v,t) = {\textsf {b}}$, then $L(v,t) \preceq L^{\prime }(v,t)$ because ${\textsf {b}}$ is the infimum of $({\mathcal {S}},\preceq )$, as indicated in the Hasse diagram to the right.
  - If  then . If not, $L^{\prime }(v,t)$ must be ${\textsf {b}}$, implying that $L^{\prime }(v^+_i,t)$ with $1\le i\le n$ and $L^{\prime }(v^-,t)$ are all ${\textsf {b}}$ by the definition of $\sqcup$. By the induction hypothesis, we further conclude that for all $i\in \{1, \ldots , n\}$ it holds that $L(v^+_i,t) = L(v^-,t) = {\textsf {b}}$, leading to a contradiction.
  - If $L(v,t) = {\textsf {a}}$ then, by the induction hypothesis, $L^{\prime }(v^+_i,t)$ (where $1\le i\le n$) and $L^{\prime }(v^-,t)$ are either ${\textsf {a}}$ or $\bot$. In all cases, the lemma holds.

$$\square $$

### **Theorem 3**

If *L* and $L^{\prime }$ are labelling functions for an (*A*, *B*)-refutation *R* (*R* being a hyper-resolution proof) and $L \preceq L^{\prime }$ such that ${\mathsf {Itp}}_i(L,R)$ as well as ${\mathsf {Itp}}_i(L^{\prime },R)$ are defined, then ${\mathsf {Itp}}_i(L,R) \Rightarrow {\mathsf {Itp}}_i(L^{\prime },R)$ (for a fixed $i\in \{1,2\}$).

### *Proof*

We prove Theorem 3 by structural induction over *R*. For any vertex *v* in *R*, let $I_v$ and $I^{\prime }_v$ be the partial interpolants due to ${\mathsf {Itp}}(L,R)$ and ${\mathsf {Itp}}(L^{\prime },R)$, respectively. We show that  for all vertices *v*, where

This establishes $I_v\Rightarrow I_v^{\prime }$ for the sink to show that ${\mathsf {Itp}}(L,R) \Rightarrow {\mathsf {Itp}}(L^{\prime },R)$.

*Base case* Let *v* be an initial vertex and let $\ell _R(v)=C$.

1. If $C\in A$, then $I_v=C\downharpoonright _{{\textsf {b}},L}$ and $I^{\prime }_v=C\downharpoonright _{{\textsf {b}},L^{\prime }}$. We need to show that . For any $t\in C\downharpoonright _{{\textsf {b}},L}$, if $L(v,t)\ne L^{\prime }(v,t)$ then , and therefore . Otherwise, $L(v,t)=L^{\prime }(v,t)={\textsf {b}}$, and therefore $t\in C\downharpoonright _{{\textsf {b}},L^{\prime }}$.
2. If $C\in B$, then $I_v = \lnot (C\downharpoonright _{{\textsf {a}},L})$ and $I^{\prime }_v = \lnot (C\downharpoonright _{{\textsf {a}},L^{\prime }})$. The proof that  is analogous to the first case.

*Induction step* We first prove a useful intermediate result. Let *v* be an internal vertex of *R* with ancestors $\{v^+_1, \ldots , v^+_n, v^-\}$. We claim that

5

for arbitrary locality preserving labelling functions *L* and $L^{\prime }$. We prove (5) by showing that any *t* contained in clause of the left-hand side of the implication must also be contained in . This holds because for any $u\in \{v^+_1, \ldots , v^+_n, v^-\}$ and $t\in \ell (u)$ with ${\mathrm {var}}(t)\ne x_i$ (for $1\le i\le n$) if  then $L(v,t)\sqcup L^{\prime }(v,t)={\textsf {a\!b}}$ since, according to Definition 1, $L(v,t)=L(v^+_1,t)\sqcup \cdots \sqcup L(v^+_n,t)\sqcup L(v^-,t)$ and $L^{\prime }(v,t)=L^{\prime }(v^+_1,t)\sqcup \cdots \sqcup L^{\prime }(v^+_n,t)\sqcup L^{\prime }(v^-,t)$.

For the induction step, let *v* be an internal vertex in *R* and let $\ell _R(v^+_i)=(x_i\vee C_i)$ and $\ell _R(v^-)=(\overline{x}_1\vee \cdots \vee \overline{x}_n\vee D)$, and let $\ell _R(v)=\bigvee _{i=1}^n C_i\vee D$. Partial interpolants are indicated as before.

*Induction hypothesis*

Recall from the proof of Lemma 1, that if $L(v^+_i,x)\preceq L^{\prime }(v^+_i,x)$ (for all $i\in \{1, \ldots , n\}$) and $L(v^-,\overline{x})\preceq L^{\prime }(v^-,\overline{x})$, then,

6 $$\begin{aligned}&L(v^+_1,x)\sqcup \cdots \sqcup L(v^+_n,x)\sqcup L(v^-,\overline{x})\quad \preceq \nonumber \\&\quad L^{\prime }(v^+_1,x)\sqcup \cdots \sqcup L^{\prime }(v^+_n,x)\sqcup L^{\prime }(v^-,\overline{x}). \end{aligned}$$

For the induction step, let $\ell (v^+_i) = (x_i \vee C_i)$ and $\ell (v^-) = (\overline{x}_1\vee \cdots \vee \overline{x}_n \vee D)$. We assume that $\forall i\in \{1, \ldots , n\}\,\cdot \,L(v^+_i, x_i)\sqcup L(v^-, \overline{x}_i)={\textsf {c}}$ (for a fixed ${\textsf {c}}$) and perform a case split on ${\textsf {c}}$. *I* and $I^{\prime }$ denote the partial interpolants due to ${\mathsf {Itp}}(L,R)$ and ${\mathsf {Itp}}(L^{\prime },R)$, respectively.

1. $\forall i\in \{1, \ldots , n\}\,\cdot \,L(v^+_i, x_i)\sqcup L(v^-, \overline{x}_i)={\textsf {a}}$: Then $I_v=\bigvee _{i=1}^{n}I_{v^+_i}\vee I_{v^-}$, and from (6) we conclude that for all $i\in \{1, \ldots , n\}$ it holds that $L^{\prime }(v^+_i,x_i)\sqcup L^{\prime }(v^-,\overline{x}_i)={\textsf {a}}$, and therefore $I^{\prime }_v=\bigvee _{i=1}^n I^{\prime }_{v^+_i}\vee I^{\prime }_{v^-}$. Moreover, $L^{\prime }(v^+_i,x_i)=L^{\prime }(v^-,\overline{x}_i)={\textsf {a}}$ for $1\le i\le n$, and therefore, the induction hypothesis can be simplified to
  We derive , and by applying (5), we obtain , which is equivalent to .
2. : We distinguish two cases for $AB$-HyRes.

In the first case, $I_v=\bigwedge _{i=1}^n(x_i\vee I_{v^+_i}) \wedge (I_{v^-}\vee \bigvee _{i=1}^n\overline{x}_i)$, and by applying the induction hypothesis we derive

Note that the right-hand side of this implication is equivalent to

which in turn implies

By applying (5), we obtain

which establishes  for the case in which  for all $i\in \{1, \ldots , n\}$. Moreover, since $\bigwedge _{i=1}^n(x_i\vee I^{\prime }_{v^+})\wedge (\overline{x}_1\vee \cdots \vee \overline{x}_n\vee I^{\prime }_{v^-})$ implies $(\bigvee _{i=1}^n I^{\prime }_{v^+_i}\vee I^{\prime }_{v^-})$ (by hyper-resolution), it also holds that  if $L^{\prime }(v^+_i,x_i)\sqcup L^{\prime }(v^-,\overline{x}_i)={\textsf {a}}$ (for $1\le i\le n$) and $I^{\prime }_v=(\bigvee _{i=1}^n I^{\prime }_{v^+_i}\vee I^{\prime }_{v^-})$. From (6) we conclude that $\not \exists i\,\cdot \,L^{\prime }(v^+_i,x)\sqcup L^{\prime }(v^-,\overline{x}_i)={\textsf {b}}$.

In the second case, $I_v=\bigvee _{i=1}^n(\overline{x}_i\wedge I_{v^+_i}) \vee (I_{v^-}\wedge \bigwedge _{i=1}^n x_i)$, and by applying the induction hypothesis we derive

which implies

and by (5) we derive , which establishes the correctness of our claim for the case in which  for all $i\in \{1, \ldots , n\}$. Moreover, since $\bigvee _{i=1}^n(\overline{x}_i\wedge I^{\prime }_{v^+})\vee (x_1\wedge \cdots \wedge x_n\wedge I^{\prime }_{v^-})$ implies $(\bigvee _{i=1}^n I^{\prime }_{v^+_i}\vee I^{\prime }_{v^-})$, it also holds that  if $L^{\prime }(v^+_i,x_i)\sqcup L^{\prime }(v^-,\overline{x}_i)={\textsf {a}}$ (for $1\le i\le n$) and $I^{\prime }_v=(\bigvee _{i=1}^n I^{\prime }_{v^+_i}\vee I^{\prime }_{v^-})$. As previously, $\not \exists i\,\cdot \,L^{\prime }(v^+_i,x)\sqcup L^{\prime }(v^-,\overline{x}_i)={\textsf {b}}$ holds.

3. $\forall i\in \{1, \ldots , n\}\,\cdot \,L(v^+_i, x_i)\sqcup L(v^-, \overline{x}_i)={\textsf {b}}$: Then $I_v=\bigwedge _{i=1}^n I_{v^+_i}\wedge I_{v^-}$. By (6), we need to distinguish three cases:
  - $\forall i\in \{1, \ldots , n\}.L^{\prime }(v^+_i,x_i)\sqcup L^{\prime }(v^-,\overline{x}_i)={\textsf {b}}$: Then, as in case 1, the induction hypothesis can be simplified to  (where $1\le i\le n$) and , and we obtain
    By an argument similar to the one made in case 2 we derive .
  - : Then $I^{\prime }_v=\bigwedge _{i=1}^n(x_i\vee I^{\prime }_{v^+_i})\wedge (\overline{x}_1\vee \cdots \vee \overline{x}_n\vee I^{\prime }_{v^-})$ in the first case of $AB$-HyRes, and by applying the induction hypothesis we derive
    The right-hand side of the implication in turn implies
    7
    and by further weakening (7) and applying (5) we derive
    8
    Finally, (8) also establishes  for case 2 of $AB$-HyRes (by Proposition 1).
  - $\forall i\in \{1, \ldots , n\}.L^{\prime }(v^+_i,x_i)\sqcup L^{\prime }(v^-,\overline{x}_i)={\textsf {a}}$: Then $I^{\prime }_v=(\bigvee _{i=1}^n I^{\prime }_{v^+_i}\vee I^{\prime }_{v^-})$. Since we have previously shown (8) and since furthermore $\bigwedge _{i=1}^n(x_i\vee I^{\prime }_{v^+_i})\wedge (\overline{x}_1\vee \cdots \vee \overline{x}_n\vee I^{\prime }_{v^-})$ implies (by Proposition 1) $\bigvee _{i=1}^n(\overline{x}_i\vee I^{\prime }_{v^+_i})\vee (x_1\wedge \cdots \wedge x_n\wedge I^{\prime }_{v^-})$, which in turn implies $\bigvee _{i=1}^n I^{\prime }_{v^+_i}\vee I^{\prime }_{v^-}$, we conclude that .

$$\square $$

### **Theorem 4**

(Correctness) For any (*A*, *B*)-refutation *R* (where *R* is a clausal proof) and locality preserving labelling function *L*, ${\mathsf {Itp}}(L,R)$ (if defined) is an interpolant for (*A*, *B*).

### *Proof*

Analogous to the proof of Theorem 1 by induction over the structure of the (*A*, *B*)-refutation *R*. Let *I* be the partial interpolant at a vertex *v* labelled with a clause $C = \ell (v)$. We show that every such *I* and *C* satisfy the following conditions:

1. $$A\wedge \lnot (C\upharpoonright _{{\textsf {a}},L})\Rightarrow I$$,
2. $$B\wedge \lnot (C\upharpoonright _{{\textsf {b}},L})\Rightarrow \lnot I$$, and
3. $$\text {Var}(I)\subseteq \text {Var}(A)\cap \text {Var}(B)$$.

For the sink *v* with $\ell (v)=\Box$, this establishes Theorem 4. The labelling function *L*, being unique in this proof, is omitted from subscripts.

*Base case* As in the proof of Theorem 1.

*Induction step* We perform a case split for the labelling of the pivots:

1. ($$A$$-TCRes): $$\forall i\in \{1, \ldots , n\}\,\cdot \,t\in P_i \Rightarrow L(v_i,t)={\textsf {a}} $$
  *Induction hypothesis* (for $i \in \{1, \ldots , n\})$:
  $$\begin{aligned} A \wedge \lnot (P_i \upharpoonright _{{\textsf {a}}}) \wedge \lnot (C_i \upharpoonright _{{\textsf {a}}})&\Rightarrow I_i\\ B \wedge \lnot (C_i \upharpoonright _{{\textsf {b}}})&\Rightarrow \lnot I_i \end{aligned}$$
  In this case $P_i \upharpoonright _{{\textsf {a}}} = P_i$, so it follows that $A \wedge \lnot (C_i \upharpoonright _{{\textsf {a}}}) \Rightarrow (P_i \vee I_i)$ and therefore:
  $$\begin{aligned} A \wedge \bigwedge _{i=1}^n \lnot (C_i \upharpoonright _{{\textsf {a}}}) \Rightarrow \bigwedge _{i=1}^n (P_i \vee I_i) \end{aligned}$$
  Since $\bigwedge _{i=1}^n P_i$ is unsatisfiable, we can apply chain resolution to the right and side to derive $\bigwedge _{i=1}^n (P_i \vee I_i) \equiv \bigvee _{i=1}^n I_i$. By furthermore rewriting the left hand side, we obtain
  $$\begin{aligned} A \wedge \lnot \left( \bigvee _{i=1}^n C_i\right) \upharpoonright _{{\textsf {a}}} \Rightarrow \bigvee _{i=1}^n I_i, \end{aligned}$$
  satisfying the first condition. Similarly, we derive
  $$\begin{aligned} B \wedge \bigwedge _{i=1}^n \lnot (C_i \upharpoonright _{{\textsf {b}}}) \Rightarrow \bigwedge _{i=1}^n \lnot I_i \quad \text {and}\quad B \wedge \lnot \left( \bigvee _{i=1}^n C_i\right) \upharpoonright _{{\textsf {b}}} \Rightarrow \lnot \bigvee _{i=1}^n I_i \end{aligned}$$
  from the induction hypothesis, which satisfies the second condition. The third condition is satisfied because for all $i \in \{1, \ldots , n\}, {\mathrm {Var}}(I_i) \subseteq {\mathrm {Var}}(A) \cap {\mathrm {Var}}(B)$ and therefore ${\mathrm {Var}}(\bigvee _{i=1}^n I_i) \subseteq {\mathrm {Var}}(A) \cap {\mathrm {Var}}(B)$ holds as well.
2. ($B$-TCRes): $\forall i\in \{1, \ldots , n\}\,\cdot \,t\in P_i \Rightarrow L(v_i, t)={\textsf {b}}$: The proof is symmetric to the first case.
3. ($$AB$$-TCRes): :
  Note that for an arbitrary labelling function *L*, we have $\lnot (P_i \upharpoonright _{{\textsf {a}}})\Rightarrow \lnot P_i$ and $\lnot (P_i \upharpoonright _{{\textsf {b}}})\Rightarrow \lnot P_i$. Therefore, we can strengthen the induction hypotheses to
  $$\begin{aligned} A \wedge \lnot P_i \wedge \lnot (C_i \upharpoonright _{{\textsf {a}}})&\Rightarrow I_i\\ B \wedge \lnot P_i \wedge \lnot (C_i \upharpoonright _{{\textsf {b}}})&\Rightarrow \lnot I_i \end{aligned}$$
  It follows immediately that
  $$\begin{aligned} A \wedge \lnot \left( \bigvee _{i=1}^n C_i \upharpoonright _{{\textsf {a}}}\right) \Rightarrow \bigwedge _{i=1}^n (P_i \vee I_i), \end{aligned}$$
  establishing the first condition for case 1 of $AB$-TCRes. By applying Proposition 2 to weaken the right hand side (which yields $A \wedge \lnot (\bigvee _{i=1}^n C_i \upharpoonright _{{\textsf {a}}}) \Rightarrow \bigvee _{i=1}^n (\lnot P_i \wedge I_i)$), we establish the condition 1 for case 2 of $AB$-TCRes.
  Analogously we derive
  9 $$\begin{aligned} B \wedge \lnot \left( \bigvee _{i=1}^n C_i \upharpoonright _{{\textsf {b}}}\right) \Rightarrow \bigwedge _{i=1}^n (P_i \vee \lnot I_i), \end{aligned}$$
  which establishes the second condition for case 2 of $AB$-TCRes ($B \wedge \lnot \left( \bigvee _{i=1}^n C_i \upharpoonright _{{\textsf {b}}}\right) \Rightarrow \lnot \bigvee _{i=1}^n (\lnot P_i \wedge I_i)$). By Proposition 2, $\lnot \bigvee _{i=1}^n (\lnot P_i \wedge I_i)$ implies $\lnot \bigwedge _{i=1}^n (P_i \vee I_i)$. Therefore, it follows that
  $$\begin{aligned} B \wedge \lnot \left( \bigvee _{i=1}^n C_i \upharpoonright _{{\textsf {b}}}\right) \Rightarrow \lnot \bigwedge _{i=1}^n (P_i \vee I_i), \end{aligned}$$
  which establishes the second condition for case 1 of $AB$-TCRes. The third condition is established by the fact that all the pivot literals in $P_i$ are shared. $\square$

### **Theorem 6**

Let *R* be a local and closed (*A*, *B*)-refutation. Then we can construct a hyper-resolution refutation *H* of (*A*, *B*) and a locality preserving labelling function *L* such that for each $v\in V_R$ with $\ell _R(v)\in {\mathcal {L}}({A})\cap {\mathcal {L}}({B})$ there exists a corresponding vertex $u\in V_H$ such that ${\mathsf {Itp}}_{KV}(R) (v)\Leftrightarrow {\mathsf {Itp}}_1(L,H) (u)$.

### *Proof*

By induction over the structure of the (*A*, *B*)-refutation *R*. Let $I_v$ be the partial interpolant at a vertex *v*.

*Base case* For each initial vertex $v\in V_R$ we construct a vertex $u\in V_H$ with $\ell _H(u)=\ell _R(v)$. First, consider the case that $\ell _R(v)\in {\mathcal {L}}({A})\cap {\mathcal {L}}({B})$. We distinguish two cases:

1. $\ell _R(v)\in A$: Then $I_v=\ell _R(v)$. Let $L(u,\ell _R(v))={\textsf {b}}$, and since $\ell _H(u)\in A$ we have $I_u=I_v$.
2. $\ell _R(v)\in B$: Then $I_v=\lnot \ell _R(v)$. Let $L(u,\ell _R(v))={\textsf {a}}$, and since $\ell _H(u)\in B$ we have $I_u=I_v$.

Otherwise, $\ell _R(v)\not \in {\mathcal {L}}({A})\cap {\mathcal {L}}({B})$. Then $L(u,\ell _H(u))={\textsf {a}}$ if $\ell _R(v)\in A$ and $L(u,\ell _H(u))={\textsf {b}}$ if $\ell _R(v)\in B$, and therefore and $I_u={\mathsf {F}}$ or $I_u={\mathsf {T}}$, respectively.

*Induction step* Let $v\in V_R$ be an internal vertex such that $\ell _R(v)\in {\mathcal {L}}({A})\cap {\mathcal {L}}({B})$ and $\{v_1, \ldots , v_n\}=\pi (v)\text {-premise}(v)$, and $\ell (v_i)\in {\mathcal {L}}({A})\cap {\mathcal {L}}({B})$ for $1\le i\le m \le n$ and $\ell (v_j)\not \in {\mathcal {L}}({A})\cap {\mathcal {L}}({B})$ for $m<j\le n$.

*Induction hypothesis* There are $\{u_1, \ldots , u_n\}\subseteq V_H$ such that

1. $$\ell _H(u_i)=\ell _R(v_i)$$ for $$1\le i\le n$$, and
2. $$I_{u_i}\Leftrightarrow I_{v_i}$$ for $$1\le i \le m$$, and
3. $I_{u_j}={\mathsf {F}}$ if $\ell (v_j)\in A$ ($I_{u_j}={\mathsf {T}}$ if $\ell (v_j)\in B$, respectively) for $m< j\le n$.

By Corollary 1, the clause $C=\lnot \ell _H(u_1)\vee \cdots \vee \lnot \ell _H(u_n)\vee \ell _R(v)$ is a tautology. We distinguish two cases:

1. ($$A$$-justified) $$\pi (v)=A$$.
  Add *C* to *A* and add $w_1$ to $V_H$ such that $\ell _H(w_1)=C$. Let  for $1\le i\le m$, $L(w_1,\lnot \ell (u_j))={\textsf {a}}$ for $m<j\le n$, and $L(w_1,\ell (v))={\textsf {a}}$. Then, add $w_2$ to $V_H$ and perform the hyper-resolution step
  $$\begin{aligned} \frac{\ell _H(u_{m+1}) \quad [{\mathsf {F}} ]\quad \cdots \quad \ell _H(u_n) \quad [{\mathsf {F}} ] \quad \ell _H(w_1)\quad [{\mathsf {F}} ]}{ \ell _H(w_2)\quad [{\mathsf {F}} ]}, \end{aligned}$$
  where $\ell _H(w_2)=\ell _H(u_1)\vee \cdots \ell _H(u_m)\vee \ell _R(v)$ and $I_{w_2}={\mathsf {F}}$. Then, add *u* to $V_H$ and perform the hyper-resolution step
  $$\begin{aligned} \frac{\ell _H(u_1) \quad [I_{u_1}]\quad \cdots \quad \ell _H(u_m) \quad [I_{u_m}] \quad \ell _H(w_2)\quad [{\mathsf {F}} ]}{\ell _H(u)\quad [I_{u}]}, \end{aligned}$$
  such that $\ell _H(u)=\ell _R(v)$ and $I_u=\bigwedge _{i=1}^m (\ell _H(u_i)\vee I_{u_i})\wedge ({\mathsf {F}} \vee \bigvee _{i=1}^m \lnot \ell _H(u_i))$.
2. ($B$-justified) $\pi (v)=B$. Analogous to the first case, but $I_{w_2}={\mathsf {T}}$. $\square$

## Footnotes

1
http://fmv.jku.at/tracecheck/README.tracecheck.

2
http://fmv.jku.at/booleforce/.

3 Note that this transformation affects the whole proof, resulting in high memory usage. ${\mathsf {TCRes}}$ offers a natural way to compute interpolants for resolution chains without intermediate clauses. Alternatively, one could apply ordinary resolution iteratively on resolution chains and retain only partial interpolants at the end of a chain. We did not experimentally evaluate the latter approach.

4
http://fmv.jku.at/aiger/.

5 In particular, it is possible to choose $\pi _R$ in such a manner that *S* is the largest sub-derivation rooted at *v* in *R* such that $\ell _R(u)\in {\mathcal {L}}({A})$ for all $u\in V_S$. This corresponds to the setting in [32, Lemma 8].
